# Determinants of sub-optimal complementary feeding practices among caregivers of children aged 6–23 months in low-and middle-income countries: scoping review

**DOI:** 10.3389/fpubh.2025.1655685

**Published:** 2025-09-26

**Authors:** Maishataba Solomon Makwela, Reneilwe Given Mashaba

**Affiliations:** ^1^Department of Human Nutrition and Dietetics, Faculty of Health Sciences, University of Limpopo, Polokwane, South Africa; ^2^Dikgale Mamabolo Mothiba (DIMAMO) Population Health Research Centre, University of Limpopo, Polokwane, South Africa

**Keywords:** infant feeding education, complementary feeding education, sub-optimal complementary feeding practice, nutritional status, determinants, low and middle-income countries, scoping review, complementary feeding knowledge

## Abstract

**Background/Objectives:**

Low to middle income countries are burdened by undernutrition and malnutrition mostly affecting children aged < 2 years due to inappropriate feeding practices. Inappropriate feeding practices have been associated with irreversible damage such as stunting and cognitive delays. Therefore, this scoping review aimed to investigate factors that influence sub-optimal complementary feeding practices among caregivers of children aged 6–23 months in low- and middle-income countries. A holistic view of these factors may assist in developing models to prevent inappropriate feeding practices.

**Methods:**

The scoping review was conducted following the Preferred Reporting Items for Systematic Reviews and Meta-Analyses extension for Scoping Reviews (PRISMA-ScR) Checklist.

**Results:**

One hundred and eight (109) manuscripts were included in the review with a sample size of 1,000,028 caregiver/mother child pairs. Several themes were identified relating to factors that contribute to sub-optimal complementary feeding practices. These included social economic factors, maternal and caregiver's characteristics, child specific factors, cultural and societal influences, health and nutritional services, environment and living conditions, as well as barriers to optimal CF practice.

**Conclusions:**

This scoping review consolidated evidence from a substantial sample of more than one million mother-child pairs from different low- and middle-income countries. The sample size and diversity provide a strong, representative foundation for informing policy, practice, and future research directions. The present study highlighted that feeding practices are affected by multiple factors and that there are interlinks between determinants of sub-optimal CF. These factors of sub-optimal CF and their respective interlinks are different for different locations and should inform future intervention studies and preventative models to better address sub-optimal CF in low to middle income countries.

## 1 Introduction

Complementary feeding is the process of moving from exclusive nursing or formula feeding to solid foods usually occurring between the ages of 6 and 23 months (1). The nutritional and dietary habits that caregivers follow during the complementary feeding period are crucial in guaranteeing ideal development, avoiding malnutrition, and creating lifetime-spanning, healthy eating patterns. However, complementary feeding strategies are less than ideal in many low- and middle-income nations though, with problems ranging from delayed introduction of solid foods to insufficient dietary diversity and nutrient consumption (2).

Given the challenges faced during this period such as lack of resources and food variety (3), and the ongoing gaps in caregivers' knowledge and practices, it is urgently necessary to thoroughly investigate the elements influencing sub-optimal complementary feeding practices across various contexts to guide efficient policy and interventions (4). This is further informed by the high prevalence of malnutrition in Africa compared to other regions. For instance, there are ~165 million stunted children, and 52 million wasted children worldwide, with the highest percentages living in Asia or Sub-Saharan Africa (SSA) (5). Several studies have reported that the main contributor to persistent childhood malnutrition and poor developmental growth in this countries is sub-optimal complementary feeding (6, 7). This justifies the need to understand factors that influence sub-optimal complementary feeding in LMIC. Therefore, a scoping review is especially suited to map the current literature, spot gaps, and offer a thorough synthesis of evidence on the several elements affecting sub-optimal complementary feeding practices in different countries. This will enable a strong basis for the next studies and direct the creation of focused interventions addressing the difficulties experienced by carers in various environments. Moreover, by combining results from several settings, the review will offer ideas pertinent for legislators, medical professionals, and development organizations striving to raise child nutrition and health in particular countries. The scattered character of current studies and the several factors influencing sub-optimal complementary feeding practices highlight the need for a scoping review in this field of research to holistically review literature on this subject. Therefore, this scoping review aimed to investigate factors influencing sub-optimal complementary feeding practices among caregivers of children aged 6–23 months in low-middle income countries.

## 2 Methods

This scoping review was conducted and reported following the Preferred Reporting Item for Systematic reviews and Meta-Analyses extension for Scoping Reviews (PRISMA-ScR) Checklist (see Supplementary file). This consistent method offers a disciplined framework to guarantee the review is thorough, open, and repeatable. Following these rules aids the scoping review to map the body of current knowledge on sub-optimal complementary feeding techniques, point out areas of weakness, and give a foundation for the next studies and policy formulation. The main elements of the study design, eligibility criteria, search strategy, study selection process, and data extraction are described in this section on methodology. To ensure transparency, the authors searched multiple databases for similar scoping reviews to avoid duplication. Without such a review, the authors continued with the scoping review.

### 2.1 Eligibility criteria

The eligibility requirements were defined to cover a wide spectrum of research pertinent to sub-optimal complementary feeding practices. Research on the elements influencing sub-optimal complementary feeding had to cover caregiver knowledge, socioeconomic level, cultural practices, and health system support. To reflect the most recent advancements in this field, the review comprised studies published from inception to August 6th, 2025. Table 1 details the eligibility criteria. Studies emphasizing sub-optimal complementary feeding outside of the specified age range, studies lacking empirical data (e.g., opinion pieces or narrative reviews), and studies lacking peer-reviewed and gray literature sources were among the exclusion criteria.

**Table 1:** Eligibility criteria.

**Criteria**	**Inclusion**	**Exclusion**
Study designs	Qualitative, quantitative, and mixed-method studies; cross-sectional, longitudinal, experimental, and quasi-experimental	Opinion pieces, narrative reviews without empirical data
Population	Caregivers of children aged 6–23 months	Caregivers of children outside the 6–23-month age range
Geographical settings	Global, with a focus on low and middle-income countries, particularly Sub-Saharan Africa	High-income countries (unless for comparative analysis)
Languages	English	Non-English publications
Date range	Inception—December 2024	Studies published before 2007
Publication type	Peer-reviewed articles	Non-peer-reviewed articles, gray literature

The review included all observational and experimental spanning across all study designs. However, reviews and opinion pieces were removed. Table 1 presents detailed eligibility criteria.

### 2.2 Search strategy

Three main academic databases—PubMed, Scopus, and Web of Science—were methodically searched. These databases were selected because of their thorough coverage of social sciences, health, and nutrition research—relevant to the issue of sub-optimal complementary feeding. Combining keywords and Boolean operators, such as “complementary feeding,” “sub-optimal complementary feeding,” “infant feeding practices,” “carer knowledge,” “child nutrition,” “feeding behavior,” and “dietary diversity,” the search terms were used. The search string for PubMed, for instance, was (“complementary feeding” OR “infant feeding practices”) AND (“carer knowledge” OR “child nutrition”) AND (“dietary diversity” OR “feeding behavior”) (see Table 2). Apart from database searches, hand-searching references from papers and important publications in the field were undertaken to find pertinent studies missed by the electronic searches. Included to guarantee thorough coverage of the issue was gray literature including WHO and UNICEF reports.

**Table 2:** Search strategy.

**Database**	**Search terms/Strings**
PubMed	(“complementary feeding” OR “infant feeding practices”) AND (“caregiver knowledge” OR “child nutrition”)
Scopus	(“complementary feeding” OR “infant feeding”) AND (“socioeconomic status” OR “feeding behavior”)
Web of Science	(“complementary feeding” AND “dietary diversity”) OR (“feeding practices” AND “child health outcomes”)

### 2.3 Study selection process

Starting with the screening of titles and abstracts depending on the eligibility criteria, the process of selecting studies consisted of several phases. To guarantee consistency and lower the possibility of bias, two reviewers separately checked every study. Discrepancies were settled by conversation or by third-reviewer consultation. For studies that satisfied the initial criteria and underwent additional eligibility screening, full-text papers were obtained. Along with explanations for exclusion, the number of studies included and excluded at each level was recorded using a PRISMA flow diagram. After the database search, the results were exported to Zotero version 6.0, a bibliographic management software, where duplicates were removed.

### 2.4 Data extraction

Standardized data extraction forms created in Microsoft Excel were applied during data collecting. Key information from every study, including the author(s), year of publication, country of study, study design, sample size, and main conclusions about sub-optimal complementary feeding practices information included the kind of complementary feeding techniques under investigation, the elements driving these methods, and the results tracked. Two reviewers (RGM and MM) then looked over the obtained data for completeness and accuracy (see Table 3).

**Table 3:** Data extraction tool.

**Data point**	**Description**
Author(s)	Name(s) of the author(s)
Year of publication	The year the study was published.
Country	The country where the study was conducted
Study design	Type of study (e.g., cross-sectional, qualitative, experimental)
Sample Size	Number of participants or units of analysis
Main findings	Key results related to sub-optimal complementary feeding practices
Factors influencing practices	Socioeconomic, cultural, health system, and other factors
Outcomes measured	Nutritional outcomes, feeding behaviors, health indicators and recommendations.

### 2.5 Data synthesis

Using a narrative approach, the gathered data were synthesized to give an overview of the main ideas and trends in sub-optimal complementary feeding methods. Common obstacles to optimal feeding practices and effective interventions were identified using theme analysis across. To offer a methodical synthesis of the data, findings were arranged under categories including carer knowledge, cultural beliefs, and socioeconomic level. We employed an upset plot to better understand the interactions between the determinants of sub-optimal complementary feeding.

## 3 Results

**Figure 1:**
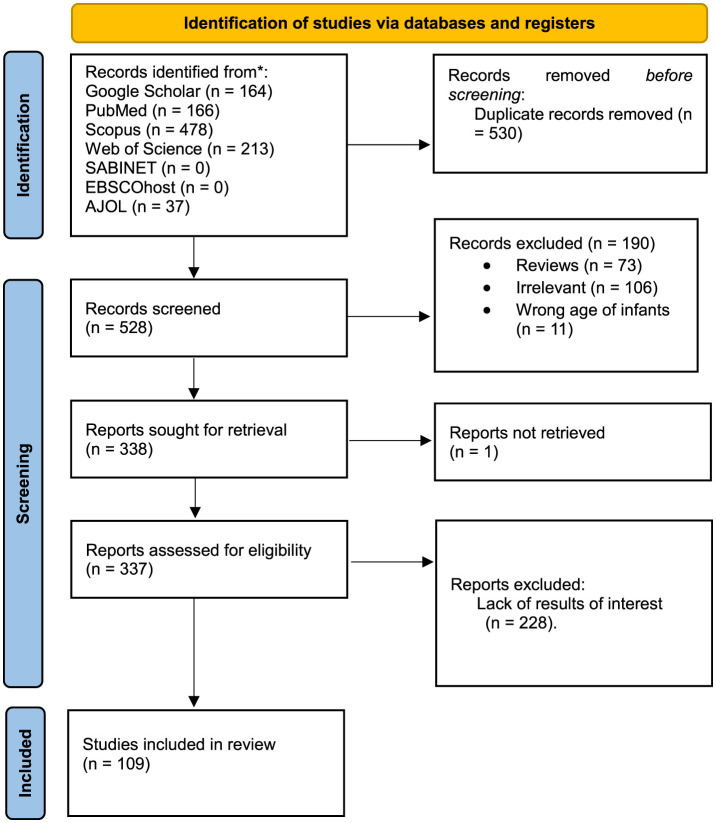
PRISMA flow diagram.

**Figure 2:**
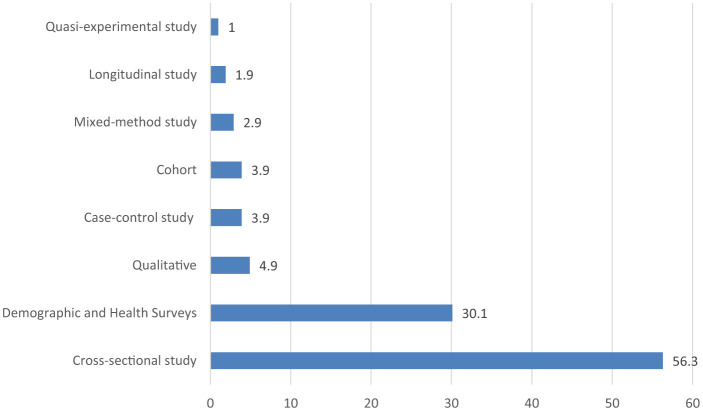
Percentage of included studies by type of study.

Figure 1 displays the study selection process using the PRISMA flow diagram. Initially, 1,058 articles were identified via electronic database searches. After removing duplicates (530), 528 records underwent inclusion screening. Among these, 190 articles were excluded based on title and abstract assessment, leaving 309 articles for eligibility assessment. Subsequently, 228 articles were excluded after a full-text examination for lack of interest results. Finally, 109 papers were included in the review (Figure 1). Supplementary Table S1 in the Supplementary File summarizes the characteristics of the articles selected for use in this review. The table clarifies the features of the articles in terms of the year of publication, the country of study, methodology, population and sample size, main results and recommendation. The included studies had 1,000,028 Caregiver/mother child pairs aged 6–23 months. Most studies included in the review were cross-sectional (56.3%), and demographic and health surveys (30.1%) followed by qualitative (4.9%), cohort (3.9%), mixed-method study (2.9%), case-control (3.9%), quasi-experimental (1%) and longitudinal (1.9%) (Figure 2).

### 3.1 Regions and countries

**Figure 3:**
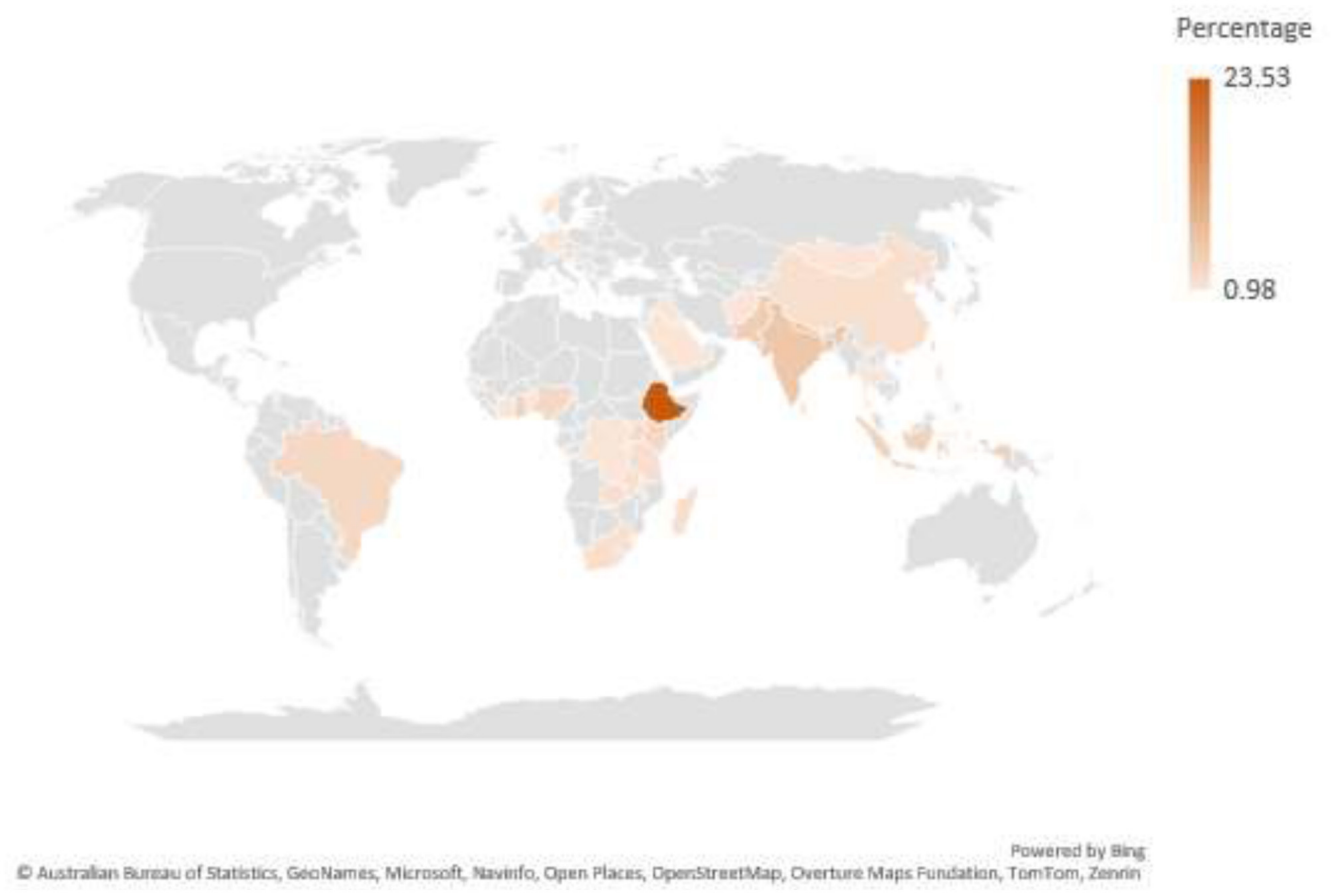
Heat Map of included studies.

**Figure 4:**
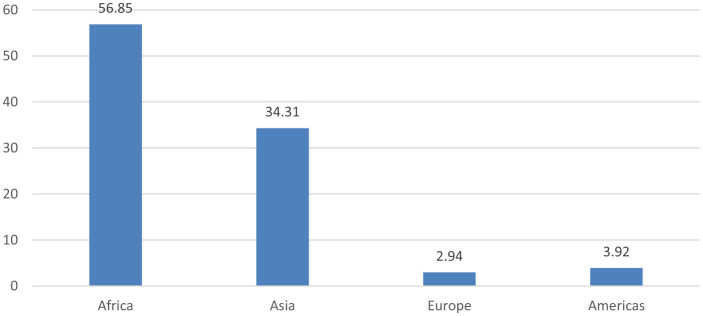
Distribution of included by region.

The majority (56.85%) of the included studies were from Africa [Ethiopia: 24, Ghana: 6, Benin: 2, Zambia: 2, Nigeria: 3, Rwanda: 2, South Africa: 2, Tanzania: 2, Malawi: 2, Côte d'Ivoire: 1, Madagascar: 2, Kenya: 1, Uganda: 2, Congo: 1, Sub-Saharan Africa: 1, Middle East and North Africa region: 1] and Asia (34.31%) [India: 5, Pakistan: 5, Bangladesh: 7, Nepal: 6, China: 2, Indonesia: 4, Sri Lanka: 1, Afghanistan: 1, Thailand: 1, Philippines: 1, Saudi Arabia: 1 and Iraq: 1 and South Asian countries: 1]. The rest of the studies were from, Europe (2.94%) [Norway: 1, Netherlands: 1 and Germany: 1] and the Americas 3.92% [Brazilian: 3 and Haiti: 1] (Figures 3, 4).

Several themes were identified relating to factors that influence sub-optimal complementary feeding practices. These included social economic factors, maternal and caregiver's characteristics, child specific factors, cultural and societal influences, health and nutritional services, environment and living conditions, as well as barriers to optimal CF practice.

### 3.2 Socioeconomic factors

Socioeconomic conditions play a significant role in shaping sub-optimal complementary feeding practices for children aged 6–24 months (4, 8–15). In households facing poverty or low wealth status, limited food options resulting from food insecurity restrict the variety/food options and nutritional quality of meals offered during this crucial stage. As a result, caregivers may prolong exclusive breastfeeding beyond 6 months, despite its declining ability to meet the child's nutritional needs (4, 8–17). At the same time, children from wealthier families are not necessarily exempt from poor feeding practices. The affordability and perceived superiority of formula milk often lead to early introduction of sub-optimal complementary foods, which is not in line with recommended guidelines (4, 8–15). The same trend has been observed at a household level where household income was found to influence the kind of food families can afford. Higher-income families were reported to be able to provide a variety of nutrient-rich options like fruits, vegetables, and fortified cereals (3, 18–27). Interestingly, families with more financial resources introduce complementary feeding too early, often influenced by aggressive marketing of formula products and afordability (3, 18–27). However, some studies reported that the cost of nutritious foods is a major barriers across all income levels. Even families with relatively stable financial situations may struggle with the high cost or limited access to healthy food options, which continues to affect the quality of children's diets during the transition from breastfeeding (28, 29).

These socio-economic factors goes beyond poverty and income but streches to issues relating to access to basic amenities such as clean water and cooking facilities (28). Caregivers find it challenging to prepare hygienic meals in places where water scarcity is prevalent, endangering the food's safety and health (30, 31). Sub-optimal complementary feeding practices feeding practices also involve parental responsibilities. For example, feeding duties are frequently delegated to other caregivers when both parents are employed, which can result in less supervision and meals that fall short of recommended standards. However, more suitable supplemental feeding has been linked to fathers' employment in particular, perhaps as a result of higher household income and support (32–34).

### 3.3 Maternal and caregiver characteristics

The characteristics of mothers and caregivers play a crucial role in shaping complementary feeding practices. There was a link between educational attainment and sub-optimal complementary feeding practices feeding knowledge (3, 6, 11, 33–41). Mothers with only elementary or secondary education were more likely to have limited knowledge about appropriate complementary feeding compared to those with higher education (3, 6, 11, 33–41). Several studies that reported that caregivers' knowledge and awareness about sub-optimal complementary feeding practices feeding, likely resulting from their educational level, are associated with better practices such as appropriate timing, food types, and feeding frequency, following guidelines (6, 14, 20, 38, 42–46). Interestingly, the education level of fathers also played a role in the feeding practice of the caregiver. Children whose fathers had at least 8 years of formal education were more likely to receive appropriate complementary feeding (3, 6, 11, 17, 33–41, 47).

The age of the mother and experience also influenced feeding practices. Young mothers (under 20 years old) were less likely to meet appropriate feeding standards compared to those aged 25 to 34 years (21, 26, 33, 48). Employment status, especially returning to work within 6 months postpartum, often affected a mother's ability to consistently implement recommended feeding practices due to the need to balance work demands with child care (26, 28, 42). These was outsite of whether the mothers were knowledgeable or not. Further more, due to work demands, caregivers are sometimes unable to attend maternal health services, and postnatal care visits thus missing out on the opportunities for health education and counseling that has the pottential to improve feeding (43–46, 49).

### 3.4 Child specific factors

Several child specific factors were found to influence how complementary feeding is practiced. For example, children older than 8 months were more likely to receive food that didn't meet recommended guidelines leading to inconsistencies in maintaining proper feeding as the child grows (13, 17, 35, 37, 43, 50–56). Secondly, caregivers were more likely to feed children who were perceived as average or larger at birth with overall better feeding practices (5, 52). Thirdly, male children were more likely to receive adequate feeding compared to females in some settings (45, 57). Lastly, having Illnesses such as respiratory infections were linked to poor feeding practices. One study found that children who were sick had the highest rates of inadequate complementary feeding, possibly due to reduced appetite or caregivers not knowing how to adjust feeding during illness (11, 32, 56–58).

### 3.5 Cultural and societal influences

Caregivers' complementary feeding practice decisions are shaped not only by what they know, but also by what they think is expected or acceptable in their community whether that's about when to start feeding, which foods to use, or even how accessible local health services are (19). Several studies noted that cultural and traditional beliefs, customs, social expectations and religion had an influence on how caregivers complementary feeding choices. For example, studies conducted in Ethiopia noted that in some communities, cultural norms favored male infants, which affects how and what female infants are fed (49, 56, 59). In addition, some studies showed that mothers who did not follow Christianity were more likely to fall short of complementary feeding recommendations, pointing to the role that religious practices or restrictions can play in shaping sub-optimal complementary feeding practices feeding choices (25, 60). Furthermore, food taboos and beliefs about certain ingredients or preparation methods were also found to play a role in caregivers complementary feeding choices often restricting what children can eat even when those foods are available and nutritious (61, 62). The custodians of cultural and traditional beliefs are generally older relatives of which several studies reported as the main source of advice for caregivers. However, these advice were reported to be shaped more by tradition than by than by current health recommendations. This affects when complementary feeding begins, what foods are given, how they're prepared, and how quickly children are introduced to the family's usual meals.

### 3.6 Health and nutrition services

Several studies reported that mothers who received guidance on breastfeeding and complementary feeding during prenatal or postnatal care visits, they were more likely to follow recommended practices (46). In contrast, mothers who missed this information were more likely to introduce complementary foods too early or too late (42, 51). Also, positive relationships with health workers helped mothers understand the benefits of appropriate feeding, and encouraged better choices for their children (26). Mothers who received information about exclusive breastfeeding and complementary feeding were more likely to follow feeding guidelines, especially if they had regular contact with under-5 clinics due to child illness (37, 52). On the other hand, those without access to this education were more likely to struggle with feeding practices.

### 3.7 Media and information exposure

Mothers who had access to media (i.e regularly watched television, listened to the radio, or had internet access) were more likely to meet the minimum dietary requirements for their children (4, 15, 29, 40, 53, 54). This is because they had access to breastfeeding promotional campaigns which had a positive influence on their feeding choices. In addition, exposure to nutrition education through community campaigns, tele health messaging, or locally produced complementary food promotions improved feeding practices and helped caregivers understand the importance of variety, frequency, and timing in infant diets (6, 12, 29, 32, 55).

### 3.8 Environmental and living conditions

Environmental and living conditions such as place of residence, availability of health facilities, and being part of a larger family/large family size were associated with sub-optimal complementary feeding (23, 54, 56–58). Children who resided in urban areas were more likely to meet complementary feeding standards in terms of dietary diversity and meal frequency compared to those in rural settings (23, 54, 56–58). However, caregivers residing in rural areas were more likely to initiate complementary feeding in a timely manner (23, 54, 56–58). Families living in areas affected by conflict or poor infrastructure were more likely to experience feeding challenges. For instance, regional factors such as agricultural practices or location within a specific ecological zone often influenced access to food and meal frequency (11, 13, 40, 59, 63, 64). Interestingly, West Africa had a higher likelihood of following appropriate feeding practices (11, 13, 40, 59, 63, 64). In areas where health services were far or limited, caregivers had less access to reliable feeding information and support, which affected how and when they introduced complementary foods (61). Larger households, low income, poor access to water, lack of cooking facilities, and limited food choices all contributed to poorer outcomes. The person who made feeding decisions in the household often influenced by gender roles also played a role, especially when mothers had little said in how income was spent or what food was bought (25, 35, 38, 54, 62).

### 3.9 Barriers to optimal practices

The most common barrier to optimal complementary feeding choice was time. Mothers often juggle caregiving with housework or employment, and this affects how much time and energy they can devote to preparing appropriate meals. In many cases, they opt for convenience thus shaping feeding decisions compromising nutritional value, especially when time was tight (60, 65). Secondly, in some homes, where male partners controlled spending, mothers had little influence over food purchases or feeding choices (21, 30, 60, 61, 66). This highlights how household power dynamics influence feeding practices. In addition, mothers who missed postnatal care were more likely to have delays or problems in starting complementary feeding, while those who did attend were more likely to meet guidelines (21, 30, 60, 61, 66). Furthermore, early introduction of complementary feeding was often associated with the belief that breast milk alone wasn't enough. Some mothers worried about milk supply, or were influenced by relatives to start feeding early, especially when babies refused new foods. These perceptions often led to suboptimal feeding practices, even when support was available (31, 48, 50, 67).

### 3.10 Interactions of determinants of sub-optimal complementary feeding practices feeding practices

**Figure 5:**
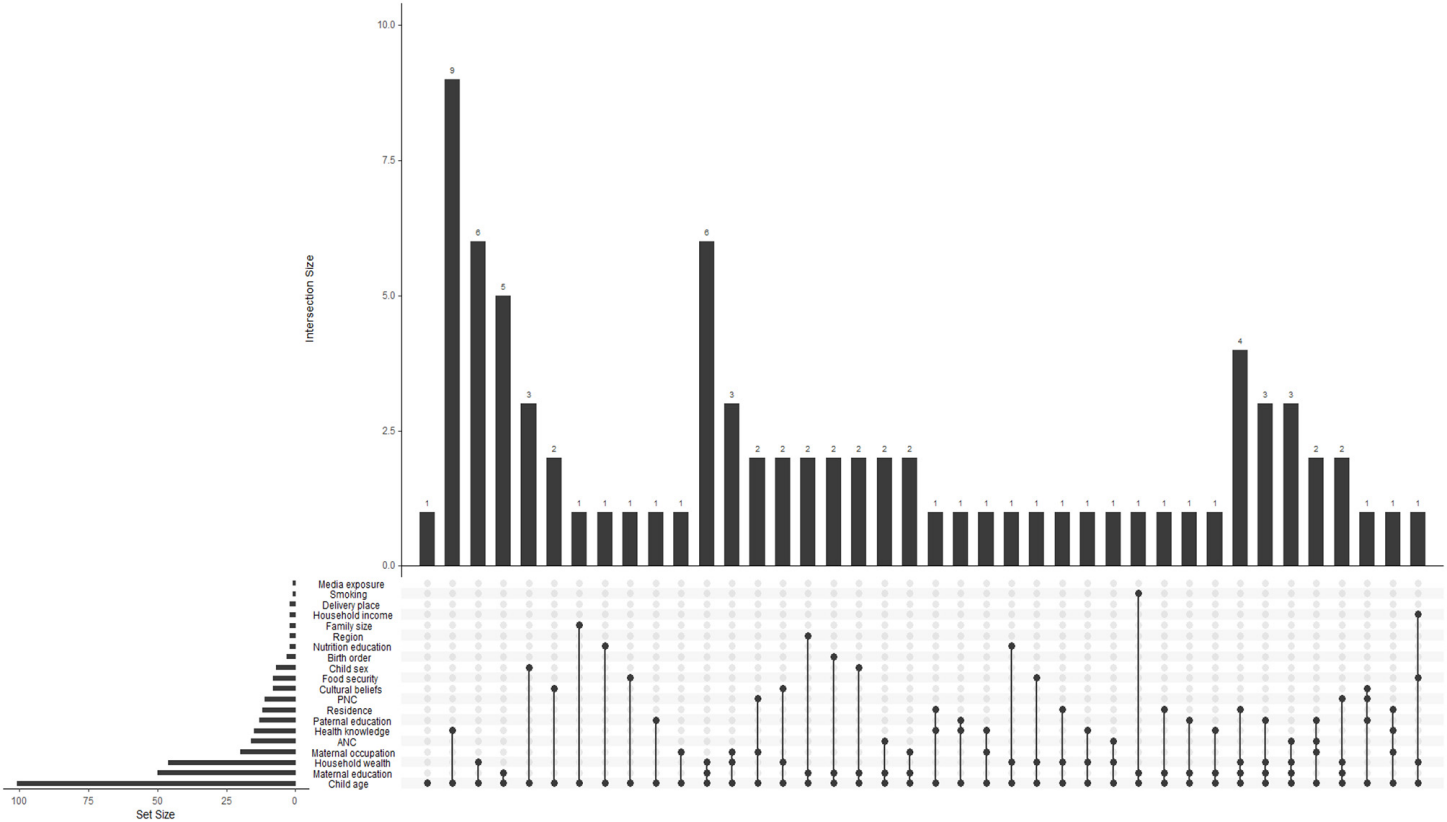
Upset plot of sub-optimal complementary feeding practices feeding determinants.

Figure 5 below is an UpSet plod that presents interactions of determinants of sub-optimal complementary feeding practices feeding practices. Firstly, the left bar plot shows how many studies reported each determinant. According to this, the largest sets of sub-optimal complementary feeding practices feeding practices determinants, reported across reviewed studies were child age, maternal education, household wealth, maternal occupation and antenatal care. Secondly, the top bar plot shows how many studies reported combinations of determinants. Thirdly, the dot matrix shows the interactions. Each column of connected dots represents a unique combination of determinants. Filled black dots indicate which determinants are involved in each combination. From this plot we found that no single determinant acts alone but a combination of determinants with overlapping influences shaped sub-optimal complementary feeding practices feeding. For instance, socioeconomic status (income, wealth, parental education), caregiver characteristics (age, occupation), and access to services (ANC, nutrition education) were frequently found together in influencing outcomes.

## 4 Discussion

This scoping review aimed to investigate factors influencing sub-optimal complementary feeding practices feeding practices and related interventions among caregivers of children aged 6–23 months in low- and middle-income countries. Several themes were identified relating to factors that influence sub-optimal complementary feeding practices feeding practices. These included social economic factors, maternal and caregiver's characteristics, cultural and societal influences, health and nutritional services, environment and living conditions, as well as barriers to optimal CF practice. For instance, socioeconomic factors were reported in most studies as the main contributor to sub-optimal CF. These socioeconomic factors included household wealth index and poverty levels (4, 8–15), food-security and household income (3, 18–27), access to basic amenities such as water and cooking facilities (30, 31), employment of both parents (32–34), and cost and accessibility of nutritious foods (28, 29). Indeed, socioeconomic factors can negatively affect CF practices. This is supported by the world health organization (WHO) report that acknowledges socioeconomic factors can be limitations and barriers to complementary feeding (68). The same report indicated the need for governments in LMIC to increase their commitments create environments that will enable families and caregivers in all circumstances to make informed choices about optimal feeding practices for infants (68). Even thou this is the case, there remains disparities in CF practices mostly affecting those with little to no economic means (67). In support of this observation, the global nutrition report reported that the prevalence of stunting is more than 2 times high in households who are poor than rich (68). This indicates that socioeconomic factors should be considered when addressing the determinants of sub-optimal complementary feeding practices feeding.

In addition to socioeconomic factors, the present review further found that caregiver specific factors contributed to sub optimal CF. This included educational attainment (3, 6, 11, 33–41), employment and work schedules (26, 28, 42), maternal age and parity (21, 26, 33, 48), knowledge and awareness about sub-optimal complementary feeding practices feeding (10, 38, 42, 43, 55, 56, 69–71). Mothers who have to return to work before 6 months after giving birth are reported to introduce CF early (21, 26, 33, 48). This is because they are not available during the day to exclusively breast feed. Furthermore, maternall time constraints and workload further affect the time they may invest in preparing complementary food for the infants (60, 65). As a result, some may opt for highly processed alternatives for convenience. Furthermore, child specific factors such as developmental stage (29, 37, 63, 70, 72–79), birth order and weight at birth (9, 76), gender (71, 80), and childhood illnesses (34, 51, 64, 81) affected the practice of CF.

Access healthcare access to health care and information plays a role in how, when and what the caregiver feed the infant (49, 61, 79). For instance, mothers who received guidance on complementary feeding during health visits and utilization such as during antenatal care, postnatal care visits (50), or through community health worker support (26), and/or access to health education and counseling (47, 49) practiced optimal CF better. Some were reported to get this information through mass media (radio, TV, internet) (4, 15, 29, 40, 53, 54) and CF promotional campaigns (6, 12, 29, 32, 55) which positively influenced CF. This suggesting that the lack of guidance and knowledge contributes to suboptimal CF. However, lack of availability of nearby health facilities may present a challenge (82). Although availability of advice has been reported to positively improve complementary feeding especially when the advice comes from trained healthcare professionals the healthcare professionals are not the only source of influence. For instance, societal customs and traditional beliefs have been purported to contribute to suboptimal CF (49, 61, 79). In addition, family members also have an influence on care givers CF choices (23, 83). Furthermore, household size and dynamics such as low household decision-making autonomy among women (21, 30, 60, 61, 66), and lack of breastfeeding support (15, 29, 42, 58, 75), contributed to early CF initiation.

### 4.1 Limitations of the study

The present review had an imbalance in terms of geographical distribution. The study included only studies published in English thus leading to some selection bias. The review included studies with different methodological approaches, a factor that may affect consistency and comparability. The exclusion of gray literature may have lead to exclusion of potentially valuable insights into the subject of sub-optimal breast feeding.

## 5 Conclusions

This scoping review consolidated evidence from a substantial sample of more than one million mother-child pairs from different low- and middle-income countries. The sample size and diversity provide a strong, representative foundation for informing policy, practice, and future research directions. The present study highlighted that feeding practices are affected by multiple factors and that there are interlinks between determinants of sub-optimal CF. These factors of sub-optimal CF and their respective interlinks are different for different locations and should inform future intervention studies and preventative models to better address sub-optimal CF in low to middle income countries.

## Data Availability

The original contributions presented in the study are included in the article/Supplementary material, further inquiries can be directed to the corresponding author.

## References

[1] Theurich MA Grote V Koletzko B. Complementary feeding and long-term health implications. Nutr Rev. (2020) 78(Supplement_2):6–12. 10.1093/nutrit/nuaa059 33196095

[2] Gatica-Domínguez G Neves PA Barros AJ Victora CG. Complementary feeding practices in 80 low-and middle-income countries: prevalence of and socioeconomic inequalities in dietary diversity, meal frequency, and dietary adequacy. J Nutr. (2021) 151:1956–64. 10.1093/jn/nxab088 33847352 PMC8245881

[3] Joshi N Agho KE Dibley MJ Senarath U Tiwari K. Determinants of inappropriate complementary feeding practices in young children in Nepal: secondary data analysis of Demographic and Health Survey 2006. Matern Child Nutr. (2012) 8(Suppl 1):45–59. 10.1111/j.1740-8709.2011.00384.x 22168518 PMC6860874

[4] Yunitasari E Al Faisal AH Efendi F Kusumaningrum T Yunita FC Chong MC. Factors associated with complementary feeding practices among children aged 6–23 months in Indonesia. BMC Pediatr. (2022) 22:727. 10.1186/s12887-022-03728-x 36539759 PMC9769005

[5] Ahmad I Khalique N Khalil S Urfi null Maroof M. Complementary feeding practices among children aged 6–23 months in Aligarh, Uttar Pradesh. J Fam Med Prim Care. (2017) 6:386–91. 10.4103/jfmpc.jfmpc_281_16 29302552 PMC5749091

[6] Ahmad A Madanijah S Dwiriani CM Kolopaking R. Determinant factors of maternal knowledge on appropriate complementary feeding of children aged 6–23 months in Aceh. J Nutr Sci Vitaminol. (2020) 66:S239–43. 10.3177/jnsv.66.S239 33612603

[7] Akanbonga S Hasan T Chowdhury U Kaiser A Akter Bonny F Lim IE . Infant and young child feeding practices and associated socioeconomic and demographic factors among children aged 6–23 months in Ghana: findings from Ghana multiple indicator cluster survey, 2017–2018. PLoS ONE. (2023) 18:e0286055. 10.1371/journal.pone.0286055 37294773 PMC10256209

[8] Debessa T Befkadu Z Darge T Mitiku A Negera E. Commercial complementary food feeding and associated factors among mothers of children aged 6–23 months old in Mettu Town, Southwest Ethiopia, 2022. BMC Nutr. (2023) 9:118. 10.1186/s40795-023-00775-0 37876015 PMC10594788

[9] Dhami MV Ogbo FA Diallo TMO Olusanya BO Goson PC Agho KE . Infant and young child feeding practices among adolescent mothers and associated factors in India. Nutrients. (2021) 13:2376. 10.3390/nu13072376 34371886 PMC8308797

[10] Gilano G Sako S Gilano K. Determinants of timely initiation of complementary feeding among children aged 6–23 months in Ethiopia. Sci Rep. (2022) 12:19069. 10.1038/s41598-022-21992-w 36351974 PMC9646799

[11] Senarath U Agho KE Akram DS Godakandage SSP Hazir T Jayawickrama H . Comparisons of complementary feeding indicators and associated factors in children aged 6-23 months across five South Asian countries. Matern Child Nutr. (2012) 8(Suppl 1):89–106. 10.1111/j.1740-8709.2011.00370.x 22168521 PMC6860856

[12] Ahmed JA Sadeta KK Lembo KH. Complementary Feeding practices and household food insecurity status of children aged 6–23 months in Shashemene City West Arsi Zone, Oromia, Ethiopia. Nurs Res Pract. (2022) 2022:9387031. 10.1155/2022/9387031 35463294 PMC9019450

[13] Akpaki K Galibois I Blaney S. Feeding practices and factors associated with the provision of iron-rich foods to children aged 6–23 months in Matam area, Senegal. Public Health Nutr. (2021) 24:4442–53. 10.1017/S1368980021002718 34284842 PMC10195257

[14] Birhanu M Abegaz T Fikre R. Magnitude and factors associated with optimal complementary feeding practices among children aged 6–23 months in Bensa District, Sidama Zone, South Ethiopia. Ethiop J Health Sci. (2019) 29:153–64. 10.4314/ejhs.v29i2.2 31011263 PMC6460456

[15] Gizaw AT Sopory P Sudhakar M. Determinants of knowledge, attitude and self-efficacy towards complementary feeding among rural mothers: baseline data of a cluster-randomized control trial in South West Ethiopia. PLoS ONE. (2023) 18:e0293267. 10.1371/journal.pone.0293267 38015909 PMC10683984

[16] Gurung TB Paudel R Anil KC Acharya A Khanal PK. Appropriate complementary feeding practice and associated factors among mothers of children aged 6–23 months in Bhimphedi rural municipality of Nepal. PloS ONE. (2024) 19:e0299969. 10.1371/journal.pone.0299969 38446802 PMC10917259

[17] Janmohamed A Luvsanjamba M Norov B Batsaikhan E Jamiyan B Blankenship JL. Complementary feeding practices and associated factors among Mongolian children 6–23 months of age. Matern Child Nutr. (2020) 16:e12838. 10.1111/mcn.12838 32835434 PMC7591305

[18] Jubayer A Nowar A Islam S Islam MdH Nayan MdM. Complementary feeding practices and their determinants among children aged 6–23 months in rural Bangladesh: evidence from Bangladesh Integrated Household Survey (BIHS) 2018–2019 evaluated against WHO/UNICEF guideline-2021. Arch Public Health. (2023) 81:114. 10.1186/s13690-023-01131-1 37344900 PMC10286447

[19] Mamo ZB Wudneh A Molla W. Determinants of complementary feeding initiation time among 6–23 months children in Gedeo Zone, South Ethiopia: community-based case-control study. Int J Afr Nurs Sci. (2022) 16:100418. 10.1016/j.ijans.2022.100418

[20] Tadesse M Ali Dawed Y Fentaw Z Endawike A Adamu K. Determinants of inappropriate complementary feeding among children 6–23 months of age in Dessie City Northeast Ethiopia: a case-control study. BMC Nutr. (2023) 9:124. 10.1186/s40795-023-00779-w 37924096 PMC10625273

[21] Aga JA Naupal-Forcadilla RT Cayetano AC. Caregivers' knowledge, attitude, and practices on complementary feeding of young children aged 6-23 months in Naga City, Philippines. J Hum Ecol Sustain. (2024) 2:11. 10.56237/jhes23004

[22] Alzaheb RA. Factors Associated with the early introduction of complementary feeding in Saudi Arabia. Int J Environ Res Public Health. (2016) 13:702. 10.3390/ijerph13070702 27420081 PMC4962243

[23] Ayu EG Gemebo TD Nane D Kuche AD Dake SK. Inappropriate complementary feeding practice and associated factors among children aged 6–23 months in Shashemene, Southern Ethiopia: a community-based cross-sectional study. BMC Pediatr. (2024) 24:573. 10.1186/s12887-024-05040-2 39251977 PMC11386105

[24] Ara R Dipti T Uddin M Ali M Rahman L. Feeding practices and its impact on nutritional status children under 2 years in a selected rural community of Bangladesh. J Armed Forces Med Coll Bangladesh. (2013) 8:26–31. 10.3329/jafmc.v8i2.1634440208441

[25] Bably MB Laditka SB Mehta A Ghosh-Jerath S Racine EF. Timing and factors associated with complementary feeding in India. Health Care Women Int. (2023) 44:220–33. 10.1080/07399332.2021.1924176 34156920

[26] Duan Y Yang Z Lai J Yu D Chang S Pang X . Exclusive breastfeeding rate and complementary feeding indicators in China: a National Representative Survey in 2013. Nutrients. (2018) 10:249. 10.3390/nu10020249 29470415 PMC5852825

[27] Mitchodigni IM Amoussa Hounkpatin W Ntandou-Bouzitou G Avohou H Termote C Kennedy G . Complementary feeding practices: determinants of dietary diversity and meal frequency among children aged 6–23 months in Southern Benin. Food Secur. (2017) 9:1117–30. 10.1007/s12571-017-0722-y

[28] Rakotomanana H Gates GE Hildebrand D Stoecker BJ. Situation and determinants of the infant and young child feeding (IYCF) indicators in Madagascar: analysis of the 2009 Demographic and Health Survey. BMC Public Health. (2017) 17:812. 10.1186/s12889-017-4835-1 29037229 PMC5644246

[29] Supthanasup A Cetthakrikul N Kelly M Sarma H Banwell C. Determinants of complementary feeding indicators: a secondary analysis of Thailand multiple indicators cluster survey 2019. Nutrients. (2022) 14:4370. 10.3390/nu14204370 36297054 PMC9610694

[30] Maciel B Moraes ML Soares AM Cruz I de Andrade M Filho JQ . Infant feeding practices and determinant variables for early complementary feeding in the first 8 months of life: results from the Brazilian MAL-ED cohort site. Public Health Nutr. (2018) 21:2462–70. 10.1017/S136898001800099X 29697043 PMC6137371

[31] Kabir A Maitrot MRL. Factors influencing feeding practices of extreme poor infants and young children in families of working mothers in Dhaka slums: a qualitative study. PLoS ONE. (2017) 12:e0172119. 10.1371/journal.pone.0172119 28207894 PMC5312963

[32] Liu J Huo J Sun J Huang J Gong W Wang O. Prevalence of complementary feeding indicators and associated factors among 6- to 23-month breastfed infants and young children in poor rural areas of China. Front Public Health. (2021) 9:691894. 10.3389/fpubh.2021.691894 34660508 PMC8517442

[33] Shumey A Demissie M Berhane Y. Timely initiation of complementary feeding and associated factors among children aged 6 to 12 months in Northern Ethiopia: an institution-based cross-sectional study. BMC Public Health. (2013) 13:1050. 10.1186/1471-2458-13-1050 24195592 PMC4228254

[34] Wasihun Y Addissie G Yigezu M Kebede N. Early initiation of complementary feeding practice and its associated factors among children aged 6 to 24 months in Northeast Ethiopia. J Health Popul Nutr. (2024) 43:67. 10.1186/s41043-024-00554-y 38755695 PMC11100127

[35] Victor R Baines SK Agho KE Dibley MJ. Factors associated with inappropriate complementary feeding practices among children aged 6–23 months in T anzania. Matern Child Nutr. (2014) 10:545–61. 10.1111/j.1740-8709.2012.00435.x 22925557 PMC6860229

[36] Abate MW Nigat AB Demelash AT Emiru TD Tibebu NS Tiruneh CM . Prevalence of timely complementary feeding initiation and associated factors among mothers having children aged 6–24 months in rural north-central Ethiopia: community based cross-sectional study. PLoS ONE. (2022) 17:e0267008. 10.1371/journal.pone.0267008 35584090 PMC9116650

[37] Belete S Kebede N Chane T Melese W Tadesse SE. Optimal complementary feeding practices and associated factors among mothers having children 6 to 23 months, south WOLLO zone, Dessie ZURIA, Ethiopia. J Pediatr Nurs. (2022) 67:e106–12. 10.1016/j.pedn.2022.08.021 36115754

[38] Gebretsadik MT Adugna DT Aliyu AD Belachew T. Optimal complementary feeding practices of children aged 6–23 months in three agro-ecological rural districts of Jimma zones of southwest Ethiopia. J Nutr Sci. (2023) 12:e40. 10.1017/jns.2023.26 37008415 PMC10052435

[39] Haile D Belachew T Berhanu G Setegn T Biadgilign S. Complementary feeding practices and associated factors among HIV positive mothers in Southern Ethiopia. J Health Popul Nutr. (2015) 34:5. 10.1186/s41043-015-0006-0 26825277 PMC5026011

[40] Mekonen EG Zegeye AF Workneh BS. Complementary feeding practices and associated factors among mothers of children aged 6 to 23 months in Sub-saharan African countries: a multilevel analysis of the recent demographic and health survey. BMC Public Health. (2024) 24:115. 10.1186/s12889-023-17629-w 38191351 PMC10775555

[41] Reda EB Teferra AS Gebregziabher MG. Time to initiate complementary feeding and associated factors among mothers with children aged 6–24 months in Tahtay Maichew district, northern Ethiopia. BMC Res Notes. (2019) 12:17. 10.1186/s13104-019-4061-2 30642371 PMC6332614

[42] Chapagain RH. Factors affecting complementary feeding practices of Nepali mothers for 6 months to 24 months children. J Nepal Health Res Counc. (2013) 11:205–7. 10.33314/jnhrc.v0i0.392 24362612

[43] Esan DT Adegbilero-Iwari OE Hussaini A Adetunji AJ. Complementary feeding pattern and its determinants among mothers in selected primary health centers in the urban metropolis of Ekiti State, Nigeria. Sci Rep. (2022) 12:6252. 10.1038/s41598-022-10308-7 35428833 PMC9012839

[44] Samuel FO Akintayo B Eyinla TE. Complementary feeding knowledge and practices of caregivers in orphanages improved after nutrition education intervention in Ibadan, Nigeria. Open J Nurs. (2021) 11:642–52. 10.4236/ojn.2021.117054

[45] Semahegn A Tesfaye G Bogale A. Complementary feeding practice of mothers and associated factors in Hiwot Fana Specialized Hospital, Eastern Ethiopia. Pan Afr Med J. (2014) 18:143. 10.11604/pamj.2014.18.143.3496 25419281 PMC4236776

[46] Umugwaneza M Havemann-Nel L Vorster HH Wentzel-Viljoen E. Factors influencing complementary feeding practices in rural and semi-urban Rwanda: a qualitative study. J Nutr Sci. (2021) 10:e45. 10.1017/jns.2021.37 34164124 PMC8190714

[47] Saaka M Awini S Nang E. Prevalence and predictors of appropriate complementary feeding practice among mothers with children 6–23 months in Northern Ghana. World Nutr. (2022) 13:14–23. 10.26596/wn.202213214-23

[48] Tromp IIM Briedé S Kiefte-de Jong JC Renders CM Jaddoe VWV Franco OH . Factors associated with the timing of introduction of complementary feeding: the Generation R Study. Eur J Clin Nutr. (2013) 67:625–30. 10.1038/ejcn.2013.50 23462942

[49] Shaker-Berbari L Qahoush Tyler V Akik C Jamaluddine Z Ghattas H. Predictors of complementary feeding practices among children aged 6–23 months in five countries in the Middle East and North Africa region. Matern Child Nutr. (2021) 17:e13223. 10.1111/mcn.13223 34137179 PMC8476411

[50] Derseh NM Shewaye DA Agimas MC Alemayehu MA Aragaw FM. Spatial variation and determinants of inappropriate complementary feeding practice and its effect on the undernutrition of infants and young children aged 6 to 23 months in Ethiopia by using the Ethiopian Mini-demographic and health survey, 2019: spatial and multilevel analysis. Front Public Health. (2023) 11:1158397. 10.3389/fpubh.2023.1158397 37965505 PMC10642280

[51] Fanta M Cherie HA. Magnitude and determinants of appropriate complementary feeding practice among mothers of children age 6–23 months in Western Ethiopia. PLoS ONE. (2020) 15:e0244277. 10.1371/journal.pone.0244277 33382749 PMC7774947

[52] Harvey S Callaby J Roberts L. An exploration of complementary feeding of infants and young children in the rural area of Muhoroni, Nyanza province, Kenya: a descriptive study. Paediatr Int Child Health. (2017) 37:172–80. 27922341 10.1080/20469047.2016.1230970

[53] Martins FA Ramalho AA de Andrade AM Opitz SP Koifman RJ de Aguiar DM . Minimum acceptable diet in a cohort of children aged between 6 and 15 months: complementary feeding assessment and associated factors in the Brazilian western Amazon. Nutr Burbank Los Angel Cty Calif. (2024) 117:112231. 10.1016/j.nut.2023.112231 37976617

[54] Nkoka O Mhone TG Ntenda PAM. Factors associated with complementary feeding practices among children aged 6–23 mo in Malawi: an analysis of the Demographic and Health Survey 2015–2016. Int Health. (2018) 10:466–79. 10.1093/inthealth/ihy047 30052967

[55] Yeshaneh A Zebene M Gashu M Abebe H Abate H. Complementary feeding practice and associated factors among internally displaced mothers of children aged 6–23 months in Amhara region, Northwest Ethiopia: a cross-sectional study. BMC Pediatr. (2021) 21:583. 10.1186/s12887-021-03050-y 34930219 PMC8686606

[56] Abebe H Gashu M Kebede A Abata H Yeshaneh A Workye H . Minimum acceptable diet and associated factors among children aged 6–23 months in Ethiopia. Ital J Pediatr. (2021) 47:215. 10.1186/s13052-021-01169-3 34717712 PMC8557568

[57] Chane T Bitew S Mekonnen T Fekadu W. Initiation of complementary feeding and associated factors among children of age 6–23 months in Sodo town, Southern Ethiopia: cross-sectional study. Pediatr Rep. (2017) 9:7240. 10.4081/pr.2017.7240 29383219 PMC5768084

[58] Dagne AH Anteneh KT Badi MB Adhanu HH Ahunie MA Tebeje HD . Appropriate complementary feeding practice and associated factors among mothers having children aged 6–24 months in Debre Tabor Hospital, North West Ethiopia, 2016. BMC Res Notes. (2019) 12:215. 10.1186/s13104-019-4259-3 30961638 PMC6454628

[59] Almeida MAM Corrente JE de Oliveira Vidal EI Gomes CB Rinaldi AEM de Barros Leite Carvalhaes MA. Patterns of complementary feeding introduction and associated factors in a cohort of Brazilian infants. BMC Pediatr. (2024) 24:629. 10.1186/s12887-024-05052-y 39358693 PMC11446015

[60] Patel A Pusdekar Y Badhoniya N Borkar J Agho KE Dibley MJ. Determinants of inappropriate complementary feeding practices in young children in India: secondary analysis of National Family Health Survey 2005–2006. Matern Child Nutr. (2012) 8(Suppl 1):28–44. 10.1111/j.1740-8709.2011.00385.x 22168517 PMC6860525

[61] Pelto GH Armar-Klemesu M. Balancing nurturance, cost and time: complementary feeding in Accra, Ghana. Matern Child Nutr. (2011) 7(Suppl 3):66–81. 10.1111/j.1740-8709.2011.00351.x 21929636 PMC6860681

[62] Issaka AI Agho KE Burns P Page A Dibley MJ. Determinants of inadequate complementary feeding practices among children aged 6–23 months in Ghana. Public Health Nutr. (2015) 18:669–78. 10.1017/S1368980014000834 24844532 PMC10271301

[63] Kurnia ID Rachmawati PD Arief YS Krisnana I Rithpho P Arifin H. Factors associated with infant and young child feeding practices in children aged 6–23 months in Indonesia: a nationwide study. J Pediatr Nurs. (2024) 78:82–8. 10.1016/j.pedn.2024.06.006 38905786

[64] Berhanu Z Alemu T Argaw D. Predictors of inappropriate complementary feeding practice among children aged 6 to 23 months in Wonago District, South Ethiopia, 2017; case control study. BMC Pediatr. (2019) 19:146. 10.1186/s12887-019-1523-6 31077158 PMC6509766

[65] Dou N Shakya E Ngoutane RM Garnier D Kouame OR Dain AL . Promising trends and influencing factors of complementary feeding practices in Côte d'Ivoire: an analysis of nationally representative survey data between 1994 and 2016. Matern Child Nutr. (2023) 19:e13418. 10.1111/mcn.13418 36069310 PMC9749586

[66] Kambale RM Ngaboyeka GA Kasengi JB Niyitegeka S Cinkenye BR Baruti A . Minimum acceptable diet among children aged 6–23 months in South Kivu, Democratic Republic of Congo: a community-based cross-sectional study. BMC Pediatr. (2021) 21:239. 10.1186/s12887-021-02713-0 34011304 PMC8132412

[67] Kegne T Alemu YM Wassie GT. Timely initiation of complementary feeding and associated factors among mothers having children aged 6 to 24 months in North-West Ethiopia: a comparative cross-sectional study. BMC Pediatr. (2024) 24:428. 10.1186/s12887-024-04906-9 38961360 PMC11223442

[68] Khanal V Sauer K Zhao Y. Determinants of complementary feeding practices among Nepalese children aged 6–23 months: findings from Demographic and Health Survey 2011. BMC Pediatr. (2013) 13:131. 10.1186/1471-2431-13-131 23981670 PMC3766108

[69] Sunuwar DR Bhatta A Rai A Chaudhary NK Tamang MK Nayaju S . The factors influencing inappropriate child feeding practices among families receiving nutrition allowance in the Himalayan region of Nepal. BMC Nutr. (2023) 9:33. 10.1186/s40795-023-00691-3 36803665 PMC9940375

[70] Dusingizimana T Weber JL Ramilan T Iversen PO Brough L. A qualitative analysis of infant and young child feeding practices in rural Rwanda. Public Health Nutr. (2021) 24:3592–601. 10.1017/S1368980020001081 32611464 PMC10195323

[71] Paul SK Roy S Islam QR Islam MZ Akteruzzaman M Rouf MA . Barriers of appropriate complementary feeding practices in under 2 children. J Bangladesh Coll Physicians Surg. (2016) 33:195–201. 10.3329/jbcps.v33i4.2813940208441

[72] Abate AD Hassen SL Temesgen MM. Timely initiation of complementary feeding practices and associated factors among children aged 6–23 months in Dessie Zuria District, Northeast Ethiopia: a community-based cross-sectional study. Front Pediatr. (2023) 11:1062251. 10.3389/fped.2023.1062251 37346895 PMC10280072

[73] Aber H Kisakye AN Babirye JN. Adherence to complementary feeding guidelines among caregivers of children aged 6–23 months in Lamwo district, rural Uganda. Pan Afr Med J. (2018) 31:17. 10.11604/pamj.2018.31.17.14955 30918545 PMC6430833

[74] Ahmed JA Sadeta KK Lenbo KH. Magnitude and factors associated with appropriate complementary feeding practice among mothers of children 6–23 months age in Shashemene town, Oromia- Ethiopia: community based cross sectional study. PLoS ONE. (2022) 17:e0265716. 10.1371/journal.pone.0265716 35349586 PMC8963544

[75] Ali M Arif M Shah AA. Complementary feeding practices and associated factors among children aged 6-23 months in Pakistan. PLoS ONE. (2021) 16:e0247602. 10.1371/journal.pone.0247602 33630931 PMC7906416

[76] Ariff S Saddiq K Khalid J Sikanderali L Tariq B Shaheen F . Determinants of infant and young complementary feeding practices among children 6–23 months of age in urban Pakistan: a multicenter longitudinal study. BMC Nutr. (2020) 6:75. 10.1186/s40795-020-00401-3 33323127 PMC7739450

[77] Bwalya R Chama-Chiliba CM Malinga S Chirwa T. Association between household food security and infant feeding practices among women with children aged 6–23 months in rural Zambia. PLoS ONE. (2023) 18:e0292052. 10.1371/journal.pone.0292052 37782631 PMC10545113

[78] Dejene Y Mezgebu GS Tadesse SE. Minimum acceptable diet and its associated factors among children aged 6-−23 months in Lalibela, northeast Ethiopia: a community-based cross-sectional study. J Nutr Sci. (2023) 12:e41. 10.1017/jns.2023.24 37123396 PMC10131047

[79] Dhami MV Ogbo FA Osuagwu UL Agho KE. Prevalence and factors associated with complementary feeding practices among children aged 6–23 months in India: a regional analysis. BMC Public Health. (2019) 19:1034. 10.1186/s12889-019-7360-6 31370827 PMC6676514

[80] Epheson B Birhanu Z Tamiru D Feyissa GT. Complementary feeding practices and associated factors in Damot Weydie District, Welayta zone, South Ethiopia. BMC Public Health. (2018) 18:419. 10.1186/s12889-018-5245-8 29587689 PMC5872387

[81] Erasmus CR Pillay T Siwela M. Factors affecting the choices made by primary caregivers during the complementary feeding transition period, KwaZulu-Natal, South Africa. South Afr J Clin Nutr. (2023) 36:1–7. 10.1080/16070658.2022.2033470

[82] Goudet SM Faiz S Bogin BA Griffiths PL. Pregnant women's and community health workers' perceptions of root causes of malnutrition among infants and young children in the slums of Dhaka, Bangladesh. Am J Public Health. (2011) 101:1225–33. 10.2105/AJPH.2010.300090 21653248 PMC3110238

[83] Heidkamp RA Ayoya MA Teta IN Stoltzfus RJ Marhone JP. Complementary feeding practices and child growth outcomes in Haiti: an analysis of data from Demographic and Health Surveys. Matern Child Nutr. (2015) 11:815–28. 10.1111/mcn.12090 24118777 PMC6860238

[84] Hazir T Senarath U Agho K Akram DS Kazmi N Abbasi S . Determinants of inappropriate timing of introducing solid, semi-solid or soft food to infants in Pakistan: secondary data analysis of Demographic and Health Survey 2006–2007. Matern Child Nutr. (2012) 8(Suppl 1):78–88. 10.1111/j.1740-8709.2011.00383.x 22168520 PMC6860553

[85] Liaqat P Rizvi MA Qayyum A Ahmed H. Association between complementary feeding practice and mothers education status in Islamabad. J Hum Nutr Diet Off J Br Diet Assoc. (2007) 20:340–4. 10.1111/j.1365-277X.2007.00791.x 17635311

[86] Harvey S Callaby J Roberts L. <no title>. (2017) 10.1080/20469047.2016.1230970 27922341

[87] Na M Aguayo VM Arimond M Dahal P Lamichhane B Pokharel R . Trends and predictors of appropriate complementary feeding practices in Nepal: an analysis of national household survey data collected between 2001 and 2014. Matern Child Nutr. (2018) 14(Suppl 4):e12564. 10.1111/mcn.12564 29148183 PMC6586161

[88] Helle C Hillesund ER Øverby NC. Timing of complementary feeding and associations with maternal and infant characteristics: a Norwegian cross-sectional study. PLoS ONE. (2018) 13:e0199455. 10.1371/journal.pone.0199455 29949644 PMC6021099

[89] Scarpa G Berrang-Ford L Twesigomwe S Kakwangire P Galazoula M Zavaleta-Cortijo C . Socio-economic and environmental factors affecting breastfeeding and complementary feeding practices among Batwa and Bakiga communities in south-western Uganda. PLoS Glob Public Health. (2022) 2:e0000144. 10.1371/journal.pgph.0000144 36962281 PMC10021580

[90] Rebhan B Kohlhuber M Schwegler U Koletzko BV Fromme H. Infant feeding practices and associated factors through the first 9 months of life in Bavaria, Germany. J Pediatr Gastroenterol Nutr. (2009) 49:467–73. 10.1097/MPG.0b013e31819a4e1a 19581814

[91] Walters CN Rakotomanana H Komakech JJ Stoecker BJ. Maternal determinants of optimal breastfeeding and complementary feeding and their association with child undernutrition in Malawi (2015–2016). BMC Public Health. (2019) 19:1503. 10.1186/s12889-019-7877-8 31711452 PMC6849257

[92] Na M Aguayo VM Arimond M Mustaphi P Stewart CP. Predictors of complementary feeding practices in Afghanistan: analysis of the 2015 Demographic and Health Survey. Matern Child Nutr. (2018) 14(Suppl 4):e12696. 10.1111/mcn.12696 30499256 PMC6587761

[93] Markos M Samuel B Challa A. Minimum acceptable diet and associated factors among 6-23 months old children enrolled in outpatient therapeutic program in the Tulla district, Sidama region, Ethiopia: a community-based cross-sectional study. J Health Popul Nutr. (2024) 43:106. 10.1186/s41043-024-00581-9 38978134 PMC11232196

[94] Al-Samarrai MAM Yaseen SM Jadoo SAA. Knowledge, attitude, and practice of mothers about complementary feeding for infants aged 6–12 months in Anbar Province, Iraq. J Ideas Health. (2020) 3:125–9. 10.47108/jidhealth.Vol3.Iss1.17

[95] Na M Aguayo VM Arimond M Narayan A Stewart CP. Stagnating trends in complementary feeding practices in Bangladesh: an analysis of national surveys from 2004–2014. Matern Child Nutr. (2018) 14(Suppl 4):e12624. 10.1111/mcn.12624 29999230 PMC6586058

[96] Ng CS Dibley MJ Agho KE. Complementary feeding indicators and determinants of poor feeding practices in Indonesia: a secondary analysis of 2007 Demographic and Health Survey data. Public Health Nutr. (2012) 15:827–39. 10.1017/S1368980011002485 22014663

[97] Issaka AI Agho KE Ezeh OK Renzaho AM. Population-attributable risk estimates for factors associated with inappropriate complementary feeding practices in The Gambia. Public Health Nutr. (2017) 20:3135–44. 10.1017/S1368980017002014 28847321 PMC10261515

[98] Senarath U Godakandage SSP Jayawickrama H Siriwardena I Dibley MJ. Determinants of inappropriate complementary feeding practices in young children in Sri Lanka: secondary data analysis of Demographic and Health Survey 2006–2007. Matern Child Nutr. (2012) 8(Suppl 1):60–77. 10.1111/j.1740-8709.2011.00375.x 22168519 PMC6860785

[99] Kabir I Khanam M Agho KE Mihrshahi S Dibley MJ Roy SK. Determinants of inappropriate complementary feeding practices in infant and young children in Bangladesh: secondary data analysis of Demographic Health Survey 2007. Matern Child Nutr. (2012) 8(Suppl 1):11–27. 10.1111/j.1740-8709.2011.00379.x 22168516 PMC6860519

[100] Bodjrènou FSU Amoussa Hounkpatin W Termote C Dato G Savy M. Determining factors associated with breastfeeding and complementary feeding practices in rural Southern Benin. Food Sci Nutr. (2021) 9:135–44. 10.1002/fsn3.1971 33473277 PMC7802539

[101] Rakotomanana H Hildebrand D Gates GE Thomas DG Fawbush F Stoecker BJ. Maternal knowledge, attitudes, and practices of complementary feeding and child undernutrition in the Vakinankaratra Region of Madagascar: a mixed-methods study. Curr Dev Nutr. (2020) 4:nzaa162. 10.1093/cdn/nzaa162 33274306 PMC7695809

[102] Kassa T Meshesha B Haji Y Ebrahim J. Appropriate complementary feeding practices and associated factors among mothers of children age 6–23 months in Southern Ethiopia, 2015. BMC Pediatr. (2016) 16:131. 10.1186/s12887-016-0675-x 27542833 PMC4992197

[103] Na M Aguayo VM Arimond M Stewart CP. Risk factors of poor complementary feeding practices in Pakistani children aged 6–23 months: a multilevel analysis of the Demographic and Health Survey 2012–2013. Matern Child Nutr. (2017) 13:e12463. 10.1111/mcn.12463 29032630 PMC6866181

[104] Ogbo FA Ogeleka P Awosemo AO. Trends and determinants of complementary feeding practices in Tanzania, 2004–2016. Trop Med Health. (2018) 46:40. 10.1186/s41182-018-0121-x 30479557 PMC6247732

