# Supplementary material for: Determinants of sub-optimal complementary feeding practices among caregivers of children aged 6–23 months in low-and middle-income countries: scoping review
Source: Front Public Health. 2025 Sep 26;13:1655685. doi: 10.3389/fpubh.2025.1655685 (PMC12511044; doi:10.3389/fpubh.2025.1655685)
Supplement: Supplementary file 1 [file Supplementary_file_1.docx]

**Supplementary document**

Determinants of sub optimal complementary feeding practices among caregivers of children aged 6–23 months in low- and middle-income countries: a scoping review

Authors: Maishataba Solomon Makwela ^1^, and Reneilwe Given Mashaba ^2,^ *

^1^ Department of Human Nutrition and Dietetics, Faculty of Health Sciences, University of Limpopo, Polokwane 0727, South Africa; [maishataba.makwela@ul.ac.za](mailto:maishataba.makwela@ul.ac.za)

^2^Dikgale Mamabolo Mothiba (DIMAMO) Population Health Research Centre, University of Limpopo, Sovenga, Polokwane 0727, South Africa; [given.mashaba@ul.ac.za](mailto:given.mashaba@ul.ac.za)

*****Correspondence: [given.mashaba@ul.ac.za](mailto:given.mashaba@ul.ac.za)

**Preferred Reporting Items for Systematic reviews and Meta-Analyses extension for Scoping Reviews (PRISMA-ScR) Checklist**

| **SECTION** | **ITEM** | **PRISMA-ScR CHECKLIST ITEM** | **REPORTED ON PAGE #** |
| --- | --- | --- | --- |
| **TITLE** | | | |
| Title | 1 | Identify the report as a scoping review. | yes |
| **ABSTRACT** | | | |
| Structured summary | 2 | Provide a structured summary that includes (as applicable): background, objectives, eligibility criteria, sources of evidence, charting methods, results, and conclusions that relate to the review questions and objectives. | Yes |
| **INTRODUCTION** | | | |
| Rationale | 3 | Describe the rationale for the review in the context of what is already known. Explain why the review questions/objectives lend themselves to a scoping review approach. | Yes |
| Objectives | 4 | Provide an explicit statement of the questions and objectives being addressed with reference to their key elements (e.g., population or participants, concepts, and context) or other relevant key elements used to conceptualize the review questions and/or objectives. | yes |
| **METHODS** | | | |
| Protocol and registration | 5 | Indicate whether a review protocol exists; state if and where it can be accessed (e.g., a Web address); and if available, provide registration information, including the registration number. | Click here to enter text. |
| Eligibility criteria | 6 | Specify characteristics of the sources of evidence used as eligibility criteria (e.g., years considered, language, and publication status), and provide a rationale. | yes |
| Information sources* | 7 | Describe all information sources in the search (e.g., databases with dates of coverage and contact with authors to identify additional sources), as well as the date the most recent search was executed. | yes |
| Search | 8 | Present the full electronic search strategy for at least 1 database, including any limits used, such that it could be repeated. | yes |
| Selection of sources of evidence† | 9 | State the process for selecting sources of evidence (i.e., screening and eligibility) included in the scoping review. | yes |
| Data charting process‡ | 10 | Describe the methods of charting data from the included sources of evidence (e.g., calibrated forms or forms that have been tested by the team before their use, and whether data charting was done independently or in duplicate) and any processes for obtaining and confirming data from investigators. | yes |
| Data items | 11 | List and define all variables for which data were sought and any assumptions and simplifications made. | yes |
| Critical appraisal of individual sources of evidence§ | 12 | If done, provide a rationale for conducting a critical appraisal of included sources of evidence; describe the methods used and how this information was used in any data synthesis (if appropriate). | yes |
| Synthesis of results | 13 | Describe the methods of handling and summarizing the data that were charted. | yes |
| **RESULTS** | | | |
| Selection of sources of evidence | 14 | Give numbers of sources of evidence screened, assessed for eligibility, and included in the review, with reasons for exclusions at each stage, ideally using a flow diagram. | yes |
| Characteristics of sources of evidence | 15 | For each source of evidence, present characteristics for which data were charted and provide the citations. | yes |
| Critical appraisal within sources of evidence | 16 | If done, present data on critical appraisal of included sources of evidence (see item 12). | yes |
| Results of individual sources of evidence | 17 | For each included source of evidence, present the relevant data that were charted that relate to the review questions and objectives. | yes |
| Synthesis of results | 18 | Summarize and/or present the charting results as they relate to the review questions and objectives. | yes |
| **DISCUSSION** | | | |
| Summary of evidence | 19 | Summarize the main results (including an overview of concepts, themes, and types of evidence available), link to the review questions and objectives, and consider the relevance to key groups. | yes |
| Limitations | 20 | Discuss the limitations of the scoping review process. | yes |
| Conclusions | 21 | Provide a general interpretation of the results with respect to the review questions and objectives, as well as potential implications and/or next steps. | yes |
| **FUNDING** | | | |
| Funding | 22 | Describe sources of funding for the included sources of evidence, as well as sources of funding for the scoping review. Describe the role of the funders of the scoping review. | yes |

JBI = Joanna Briggs Institute; PRISMA-ScR = Preferred Reporting Items for Systematic reviews and Meta-Analyses extension for Scoping Reviews.

* Where *sources of evidence* (see second footnote) are compiled from, such as bibliographic databases, social media platforms, and Web sites.

† A more inclusive/heterogeneous term used to account for the different types of evidence or data sources (e.g., quantitative and/or qualitative research, expert opinion, and policy documents) that may be eligible in a scoping review as opposed to only studies. This is not to be confused with *information sources* (see first footnote).

‡ The frameworks by Arksey and O’Malley (6) and Levac and colleagues (7) and the JBI guidance (4, 5) refer to the process of data extraction in a scoping review as data charting*.*

§ The process of systematically examining research evidence to assess its validity, results, and relevance before using it to inform a decision. This term is used for items 12 and 19 instead of "risk of bias" (which is more applicable to systematic reviews of interventions) to include and acknowledge the various sources of evidence that may be used in a scoping review (e.g., quantitative and/or qualitative research, expert opinion, and policy document).

*From:* Tricco AC, Lillie E, Zarin W, O'Brien KK, Colquhoun H, Levac D, et al. PRISMA Extension for Scoping Reviews (PRISMAScR): Checklist and Explanation. Ann Intern Med. 2018;169:467–473. [doi: 10.7326/M18-0850](http://annals.org/aim/fullarticle/2700389/prisma-extension-scoping-reviews-prisma-scr-checklist-explanation).

**Table S1: Summary of characteristics of included studies**

| **Author** | **Country** | **Population and sample size** | **Methodology** | **Main results** |
| --- | --- | --- | --- | --- |
| Abate et al., 2023(1) | Ethiopia | 770 mother child pairs aged 6–23 months. | cross-sectional study | Maternal occupation, radio availability, ANC follow-up, place of delivery, and postnatal care visit. |
| Abate et al., 2022(2) | Ethiopia | 570 mother child pairs aged 6–23 months. | Cross-sectional study | Home delivery, unable to attend postnatal care follow-up, being housewife and farmers. |
| Ababe et al., 2021(3) | Ethiopia | 575 mother child pairs aged 6–23 months. | Cross-sectional study | Age of infant, fathers educational, having more than three postnatal care visits, being a housewife, being male gender, and maternal age between 25 and 34 years were some of the factors associated with the practice of minimum acceptable diet. |
| Aber et al., 2018(4) | Uganda | 349 mother child pairs aged 6–23 months. | cross-sectional study | The odds of adherence to CF were higher among caregivers with children aged 6-8 months, children whose fathers had attained 8 or more years of formal education, caregivers with two children under five years, those living in the poorest households. |
| Aga et al., 2024(5) | Philippines | 60 mother child pairs aged 6–23 months. | cross-sectional study | Educational attainment, occupation, and household income. |
| Ahmad et al., 2020(6) | Indonesia | 392 mother child pairs aged 6–23 months. | cross-sectional study | Education level of mothers. |
| Ahmad et al., 2017(7) | India | 326 mother child pairs aged 6–23 months. | cross-sectional study | Area of residence, birth order of child, and Standard of living index (SLI); MMF, sex of child, and literacy status of mother. |
| Ahmed et al., 2022(8) | Ethiopia | 536 mother child pairs aged 6–23 months. | cross-sectional study | Household socioeconomic status (wealth index, food security status, household income) and child age. |
| Ahmed et al., 2022(9) | Ethiopia | 536 mother child pairs aged 6–23 months. | cross-sectional study | Child age, socioeconomic status, mothers in the wealth index of the household, number of antenatal care visits greater, information sources. |
| Akanbonga et al., 2023(10) | Ghana | 2585 mother child pairs aged 6–23 months. | Cluster survey | Educational status of mothers/caretakers, household wealth index, administrative region and area of residence, age of IYC. |
| Akpaki et al., 2021(11) | Senegal | 98 mother child pairs aged 6–23 months. | cross-sectional study | Household food insecurity status and age of the child rather than mothers’ psychosocial factors were significant predictors of IRF consumption amongst children aged 6–23 months in the study area. |
| Al-samarraie et al., 2020(12) | Iraq | 219 mother child pairs aged 6–23 months. | cross-sectional study | The impact of family, society, and cultural background positively reflected on maternal behavior. |
| Ali et al., 2021(13) | Pakistan | 2688 mother child pairs aged 6–23 months. | demographic survey | Child’s age, weight at birth, the wealth of the household and prenatal visits at the community level. |
| Almeida et al., 2024(14) | Brazilian | 567 mother child pairs aged 6–23 months. | cross-sectional study | Having an adolescent mother or a mother over 34 years old increased the prevalence of babies in high Infant Formula and Later Ultra-processed Food Introduction, as well as being born to mothers who did not receive guidance on breastfeeding and introduction of complementary foods during prenatal care. |
| Alzaheb et al., 2016(15) | Saudi Arabia | 632 mother child pairs aged 6–23 months. | cross-sectional study | younger age; shorter education; employment within 6 months post-birth; caesareans; not breastfeeding fully for six weeks post-birth, and living in low-income households. |
| Ariff et al., 2020(16) | Pakistan | 250 mother child pairs aged 6–23 months. | longitudinal study | Maternal education, employment, and the child’s age. |
| Ayu et al., 2024(17) | Ethiopia | 609 mother child pairs aged 6–23 months. | cross-sectional study | Child’s age, maternal age, maternal occupation, and decision-making role on income. |
| Ara et al., 2013(18) | Bangladesh | 227 | Cross sectional | Mothers literacy, age, employment status, family income |
| Bably et al., 2023(19) | India | 11218 mother child pairs aged 6–23 months. | cross-sectional study | Demographic factors were associated with delaying initiating complementary feeding, including lower-income, rural residence, lower education, and religion. |
| Belete et al., 2022(20) | Ethiopia | 732 mother child pairs ged 6–23 months. | cross-sectional study | The factors affecting complementary feeding practice in NGO-supported kebeles were the child's age, educational status of the father, and hand washing latrine facility while the child's age and receiving counseling on complementary feeding practice were the only factors in not supported kebeles. |
| Berhanu et al., 2019(21) | Ethiopia | 372 mother child pairs aged 6–23 months. | Case-control | Paternal household decision making on feeding, family priority to elders during feeding, absence of nearby health facility, unplanned pregnancy, missing ANC and missing EPI service utilization. Whereas; lack of awareness, short birth spacing practice, poverty and feeding culture were community related factors |
| Birhanu et al., 2019(22) | Ethiopia | 622 mother child pairs aged 6–23 months. | cross-sectional study | Mother’s knowledge, postnatal care utilization and household food security. |
| Bodjrènou et al., 2021(23) | Benin | 360 mother child pairs aged 6–23 months. | cross-sectional study | Socioeconomic, cultural, and demographic factors such as age and sex of children, district of residence, ethnic group, education level, and employment status. |
| Bwalya et al., 2023(24) | Zambia | 2127 mother child pairs aged 6–23 months. | population suvey | Children living in households classified as being food-secure based on the household dietary diversity score were significantly more likely to achieve appropriate CF. |
| Chane et al., 2017(25) | Ethiopia | 623 mother child pairs aged 6–23 months. | cross-sectional study | Maternal illiteracy, low socioeconomic status, presence of medical illness and lack of source of information about complementary feeding practices were associated with timely initiation of complementary feeding. |
| Chapagain., 2013(26) | Nepal | 1100 mother child pairs aged 6–23 months. | cross-sectional study | Lack of knowledge regarding ideal feeding practice. Whether or not mother received feeding advice. Education of mother, type of family, profession of father, whether mother is a housewife or job holder. |
| Dagne., 2019(27) | Ethiopia | 401 mother child pairs aged 6–23 months. | cross-sectional study | Mothers’ level of education, husbands’ occupation, mothers ‘having exclusive breast feeding practice, health education about exclusive breast feeding during antenatal care visit and advice on appropriate complementary feeding practice during ANT visit, and mothers who have got under 5 unit service due to infant and young children illness were statistically significant variables for appropriate complementary feeding practice. |
| Debessa., 2023(28) | Ethiopia | 386 mother child pairs aged 6–23 months. | cross-sectional study | The age of index child 0-11months, non-exclusive breastfeeding, exposure to CCF promotions, maternal employment, and higher tertile wealth status were significantly associated with CCF feeding. |
| Dejene., 2023(29) | Ethiopia | 387 mother child pairs aged 6–23 months. | cross-sectional study | Sex of child, getting infant and young child feeding counselling at antenatal care, infant feeding practice-related knowledge and childhood illness are the variables that were found to be an independent predictor of minimum acceptable diet. |
| Derseh., 2023(30) | Ethiopia | 1463 mother child pairs aged 6–23 months. | demographic survey | Mothers with no education, poorest households, mothers who did not have post-natal checkups, non-breastfeed status and child age older than 12 months were negatively associated with IACFP. |
| Dhami et al., 2021(31) | India | 5148 mother child pairs aged 6–23 months. | cross-sectional study | Maternal education, mode of delivery, frequency of antenatal care (ANC) clinic visits, geographical region, child’s age, and household wealth were the main factors associated with breastfeeding practices while maternal education, maternal marital status, child’s age, frequency of ANC clinic visits, geographical region, and household wealth were factors associated with complementary feeding practices. |
| Dhami et al., 2019(32) | India | 69464 mother child pairs aged 6–23 months. | demographic survey | Higher household wealth index, higher maternal education, and frequent antenatal care visits (≥4 visits). |
| Dou et al., 2022(33) | Côte d'Ivoire | 6935 mother child pairs aged 6–23 months. | Demographic and Health Surveys | Older children and those from urban households had higher odds of meeting MDD and MAD. Maternal TV watching was associated with higher odds of meeting MDD. The secondary or higher education levels of mothers significantly predicted higher odds of meeting INTRO and MDD. Currently, breastfeeding was also positively associated with odds of meeting MMF and MAD. Children from poorer households had lower odds of meeting MMF, MDD and MAD. |
| Duan et al., 2018(34) | China | 14458 mother child pairs aged 6–23 months. | survey | Low household income and maternal education were potential risk factors for poor complementary feeding practice. |
| Dusingizimana et al., 2021(35) | Rwanda | 18 mother child pairs aged 6–23 months. | Qualitative | Sub-optimal complementary feeding practices result from the complex beliefs, perceptions and food classification systems which appear to have a strong influence on mothers’ feeding decisions. |
| Epheson et al., 2018(36) | Ethiopia | 401 mother child pairs aged 6–23 months. | cross-sectional study | Being a government employee mother, attending postnatal care and having a child with birth interval greater than 3 years were associated with appropriate complementary feeding. |
| Erasnus et al.,2023(37) | South Africa | 25 mother child pairs aged 6–23 months. | Qualitative | Family advice, customs and cultural belief systems, food accessibility, convenience and affordability. |
| Esan et al.,2022(38) | Nigeria | 135 mother child pairs aged 6–23 months. | cross-sectional study | Mother’s knowledge of ideal age for introduction of complementary feeding was the major factor for early CF initiation in this study. |
| Fanta et al., 2020(39) | Ethiopia | 323 mother child pairs aged 6–23 months. | cross-sectional study | Age of the child specifically lower age, birth order and mother’s education. |
| Gebretsadik et.al., 2023(40) | Ethiopia | 843 mother child pairs aged 6–23 months. | cross-sectional study | Maternal educational status, family size, agro-ecological residence and knowledge were significant predictors of OCFP. |
| Gilano et al., 2022(41) | Ethiopia | 4061 mother child pairs aged 6–23 months. | cross-sectional study | Maternal education, wealth index and shorter birth intervals contributed to poor timing of CF. The availability of formula feeding for healthy children and the promotion of such products might be attracting many families to begin early complementary feeding. |
| Gizaw et al., 2023(42) | Ethiopia | 516 mother child pairs aged 6–23 months. | cross-sectional study | Maternal educational status, the number of ANC visits, and the CF information received were predictors of CF knowledge. Likewise, child sex and the number of ANC visits were predictors of CF attitude. Family size and household food security status were predictors of CF self-efficacy |
| Goudet et al., 2011(43) | Bangladesh | 18 mothers | Qualitative | lack of knowledge, time, and resources |
| Gurung et al., 2024(44) | Nepal | 259 mother child pairs aged 6–23 months. | cross-sectional study | Factors such as spouse education, counseling on IYCF, kitchen garden, and household food security had positive association with appropriate CF practice. |
| Haile et al., 2015(45) | Ethiopia | 2015 mother child pairs aged 6–23 months. | cross-sectional study | Higher educational status of the mother was the predictor of bottle-feeding. |
| Heidkamp et al., 2015(46) | Haity | 1701 mother child pairs aged 6–23 months. | Demographic Health Survey | Mothers with secondary or more education, achieving MDD or MAD. |
| Helle et al., 2018(47) | Norway | 715 mother child pairs aged 6–23 months. | cross-sectional study | Infants of younger, less educated and smoking mothers are at higher risk of not being fed incompliance with the official infant feeding recommendations. |
| Harvey et al., 2017(48) | Kenya | 400 mother child pairs aged 6–23 months. | Coss sectional | Age of the infant |
| Hazir et al., 2012(49) | Pakistan | 941 mother child pairs aged 6–23 months. | Demographic and Health Survey | Mothers from poor household were significantly more likely to delay introduction of complementary foods. |
| Issaka et al., 2014(50) | Ghana | 822 mother child pairs aged 6–23 months. | Demographic and Health Survey | Non-attendance of postnatal check-up by mothers, cultural beliefs and habits, household poverty, home delivery of babies and non-Christian mothers were the most important risk factors for inadequate complementary feeding practices. |
| Issaka et al., 2017(51) | Gamdia | 2362 mother child pairs aged 6–23 months. | Demographic and Health Survey | Mothers aged less than 20 years, children born at home and children from poor households. |
| Janmohamed et al., 2019(52) | Mongolia | 938 mother child pairs aged 6–23 months. | Survey | Increased household wealth was positively associated with all three indicators, whereas severe food insecurity was not associated with MMF, MDD, or MAD. Older child age and maternal dietary diversity were positively associated with child MDD. |
| Joshi et al., 2012(53) | Nepal | 1428 mother child pairs aged 6–23 months. | survey | Children living in poor households were significantly less likely to meet minimum dietary diversity and minimum acceptable diet. Mothers who had adequate exposure to media, i.e. who watch television and who listen to radio almost every day, were significantly more likely to meet minimum dietary diversity and meal frequency. Infants aged 6–11 months were significantly less likely to meet minimum acceptable diet and to meet minimum meal frequency. |
| Jubayer et al., 2023(54) | Bangladesh | 665 mother child pairs aged 6–23 months. | survey | Child age, education level of mothers, ANC, household food security, monthly household income, and residential area were significantly associated with various components of CF practice. |
| Kabir et al., 2017(55) | Bangladesh | 16 mother child pairs aged 6–23 months. | qualitative | Infants and young children infamilies with working mothers are broadly determined by mothers’ occupation, basis civic facilities, and limited family buying capacity. Household composition, access to cooking facilities, and poverty level were also found tobe significant determining factors. |
| Kabir et al., 2012(56) | Bangladesh | 1728 mother child pairs aged 6–23 months. | survey | Several factors that were consistently associated with poor complementary feeding indicators including low household wealth, low levels of parental education, especially father’s education, and selected geographic areas in the country. |
| Kambale et al., 2021(57) | Congo | 742 mother child pairs aged 6–23 months. | cross-sectional study | Residence urban area, attendance postnatal care, education status of mother and household socioeconomic status were factors positively associated with minimum acceptable diet. |
| Kassa et al., 2016(58) | Ethiopia | 611 mother child pairs aged 6–23 months. | cross-sectional study | Educated mothers, older children aged 12–23 months and smaller family size were factors that can increase appropriate complementary feeding practice. |
| Kegne et al., 2024(59) | Ethiopia | 1100 mother child pairs aged 6–23 months. | cross-sectional study | Urban residence, had antenatal care visits, had post-natal care checkups and being a governmental employee were factors associated with timely initiation of CF among urban mothers. Whereas in rural settings: institutional delivery, post natal care checkups, being daily laborer were associated with timely initiation of CF. |
| Kinoti et al., 2016 (60) | Kenya | 350 mother child pairs aged 6–23 months. | cross-sectional study | Lack of awareness of the quality and quantity of food to feed the infant. Marital status of the care giver. |
| Kimiywe et al., 2015 (61) | Kenya | 201 mother child pairs aged 6–23 months. | cross-sectional study | Care givers who had low educational attainment and low educational status associated with sub optimal complementary feeding. lack of food options. |
| Khanal et al., 2013(62) | Nepal | 698 mother child pairs aged 6–23 months. | survey | ANC visits, and the place where the family lives (development region, and ecological region) were significant determinants impacting on meeting the recommended meal frequency. ANC visits and education of mothers were significant determinants of meeting the recommended minimum acceptable diet. |
| Kurnia et al., 2024(63) | Indonesia | 4943 mother child pairs aged 6–23 months. | cross-sectional study | Child's age, household wealth, regional disparities, place of residence, and history of childhood illnesses, such as fever. |
| Liaqat et al., 2007(64) | Pakistan | 500 mother child pairs aged 6–23 months. | Cross sectional | Mother's education |
| Liu et al., 2021(65) | China | 13972 mother child pairs aged 6–23 months. | cross-sectional study | Age of IYC, education level of parents, paternal employment, and nutrition knowledge of parents were positively associated factors for the prevalence of meeting requirements of MAD, and diarrhea at 2 weeks and maternal employment were negatively associated with MAD. |
| Mphasha et al., 2023(66) | South Africa | 25 mother child pairs aged 6–23 months. | Qualitative | The inability to afford food. lack of time to prepare food. misguided advices from the society about complementary feeding. Personal preferences. |
| Maciel et al., 2018(67) | Brazilian | 233 mother child pairs aged 6–23 months. | cohort | Breastfeeding initiation within the first hour of birth and higher SES were determinant variables preventing the early introduction of water and other milks, respectively. |
| Mamo et al., 2022(68) | Ethiopia | 360 mother child pairs aged 6–23 months. | Case-comtrol | Marital status such as access to water, maternal level of education, household feeding decision maker, household food insecurity, household feeding preference, family perception of breast feeding initiation time, knowledge, and perception on availability of health facilities were determinants of complementary feeding initiation practices. |
| Markos et al., 2024(69) | Ethiopia | 346 mother child pairs aged 6–23 months. | cross-sectional study | Mothers who did not have formal education, mothers who had a good knowledge of recommended feeding, and children who were aged 18–23 months were significantly associated with a minimum adequate diet |
| Martins et al., 2024(70) | Brazilian | 857 mother child pairs aged 6–15 months. | cross-sectional study | Minimum acceptable diet was inversely associated with C, D, and E social classes, number of living siblings. Maternal regular consumption of fruits, vegetables, and legumes, child age from 12 to 15 month, and receiving guidance regarding complementary feeding during postnatal consultations were directly associated with minimum acceptable diet. |
| Mekonen et al., 2024(71) | Sub Saharan Africa | 60266 mother child pairs aged 6–23 months. | survey | Higher maternal educational level, female household head, having media exposure, attending more ANC visits, health facility delivery, currently breastfeeding, having PNC follow-up, low community illiteracy, and living in the West Africa region increase the odds of appropriate complementary feeding practices. |
| Mitchodigni et al., 2017(72) | Benin | 1225 mother child pairs aged 6–23 months. | cross-sectional study | Child age, household production diversity, share of the household income allocated to food, commune of residence, ethnic group of household head, caregivers’ occupation, marital status of caregivers and household size. |
| Mokori et al., 2017 (73) | Uganda | 2958 mother child pairs aged 6–23 months. | Survey | Age of the child. Not attending post-natal care. |
| Owino et al., 2008(74) | Zambia | 34 mother child pairs aged 6–23 months. | Mixed method | High food prices. Time constrains for food preperationsespecially in mothers who are working. |
| Okafoagu et al., 2017(75) | Nigeria | 296 mother child pairs aged 6–23 months. | cross-sectional study | The age of both the infant and mother. Cultural factors of when it is appropriate to introduce solid foods. |
| Na et al., 2017(76) | Pakistan | 2827 mother child pairs aged 6–23 months. | survey | Consistent risk factors across multiple poor feeding indicators indicate the need to prioritize future interventions and programs for infants and children aged 6–11 months, whose mothers have lower or no access to prenatal and postnatal care services, from poorer households and from communities with poorer general access to health and nutrition services. |
| Na et al., 2018(77) | Afghanistan | 7963 mother child pairs aged 6–23 months. | survey | Risk factors for poor complementary feeding practices included younger age, poor access to health care services, household poverty, and residence in the Central Highland region. |
| Na et al., 2018(78) | Bangladesh | 8277 mother child pairs aged 6–23 months. | survey | Complementary feeding practices varied by individual, household, and community characteristics. Young child age (6–11 months), poor parental education, household poverty, and residence in the Chittagong and Sylhet regions independently predicted poorer feeding practices, |
| Na et al., 2018(79) | Nepal | 6205 mother child pairs aged 6–23 months. | survey | Disparities in the risk of inappropriate complementary feeding practices are significantly evident at both individual (child age, maternal education, antenatal visits, and sociocultural group) and community‐level (ecological zone and community‐level access to health care) |
| Ng et al., 2011(80) | Indonesia | 4604 mother child pairs aged 6–23 months. | survey | Infants from poor households were significantly less likely to be introduced to complementary feeding and meet the minimum dietary diversity. Mother’s education, no education, residence and decreased age of the infant were negatively associated with minimum dietary diversity, minimum meal frequency and an acceptable diet. Infants aged 6–11 months were also significantly less likely to meet minimum dietary diversity, minimum meal frequency and minimum acceptable diet. |
| Nkoka et al., 2018(81) | Malawi | 4732 mother child pairs aged 6–23 months. | Surveys | Children from mothers with secondary and from mothers working in agriculture and living in the central region, were significantly more likely to have appropriate CFP. Being over one year of age was associated with reduced odds of achieving minimum meal frequency. Children from rich households were more likely to achieve both minimum dietary diversity and minimum acceptable diet. |
| Ogbo et al., 2018(82) | Tanzania | 2949 mother child pairs aged 6–23 months. | survey | Higher maternal education and household wealth, mother’s employment, health facility birthing, PNC visits and TBA-assisted births for the introduction of complementary foods. |
| Patel et al., 2012(83) | India | 15027 mother child pairs aged 6–23 months. | survey | Factors that consistently impacted inappropriate feeding indicators were the low level of the mother’s education, lower frequency of antenatal visits and no exposure to media. |
| Pelto et al., 2011(84) | Ghana | 30 mother child pairs aged 6–23 months. | qualitative | Economic Constraints such as Cost of food, Perception of Food Healthiness. Time Management and Convenience, Time constraints Multiple demands on women’s time influence food preparation and acquisition decisions. Convenience: Implicitly important, even if not explicitly valued, shaping decisions to save time for non-food-related activities. Household dynamics: Decision-making is often influenced by relationships with other household members, especially male partners |
| Paul et al., 2016 (85) | Bangladesh | 400 mother child pairs aged 6–23 months. | Cross sectional | Mothers’ perception that breast milk alone was not enough and refusal of complementary foods by their babies, and influence by relatives. |
| Rakotomanana et al., 2017(86) | Madagascar | 1956 mother child pairs aged 6–23 months. | survey | Younger maternal age at first birth, low maternal education and low household wealth were associated with inappropriate feeding practices. |
| Rakotomanana et al.,2020(87) | Madagascar | 220 mother child pairs aged 6–23 months. | Mixed-Methods Study | Maternal attitudes about complementary foods, as well as mothers’ workload and very low income, were identified as barriers to optimal feeding practices. Maternal perceived benefits of giving appropriate complementary foods as well as their positive relationship with the community health workers were the main facilitators of optimal child feeding. |
| Rebhan et al., 2009(88) | Germany | 3103 mother child pairs aged 6–23 months. | cohortpatel | The strongest risk factor associated with complementary feeding before the fifth month was breast-feeding duration of less than 4 months. Other factors were low level of education, young age of the mother, smoking habit of the mother, and mother not being born in Germany. |
| Reda et al., 2019(89) | Ethiopia | 633 mother child pairs aged 6–23 months. | cohort | Time to initiate into CF was affected by maternal employment, husbands educational status, birth preparedness, growth monitoring, ability to know the exact time to introduce complementary feeding, and husband support. |
| Saaka et al., 2022(90) | Ghana | 1260 mother child pairs aged 6–23 months. | quasi experimental | Children aged 12-23 months, higher nutrition related knowledge of caretakers, higher educational level of mothers, and positive nutrition related attitudes towards appropriate complementary feeding practices were significantly associated with appropriate complementary feeding practice. |
| Hoche et al., 2018(91) | Ethiopia | 634 mother-infant pairs | Cross sectional | Maternal education. Family size. Fewer ANC visits. No breastfeeding counseling. |
| Samuel et al., 2021(92) | Nigeria | 50 mother child pairs aged 6–23 months. | quasi-experimental study | Nutrition education improved the complementary feeding knowledge and practice of caregivers. |
| Scarpa et al., 2022(93) | Uganda | 94 mother child pairs aged 6–23 months. | mixed-method study | Marginalization and poverty; environmental change; lack of information; and poor support. |
| Semahegn et al., 2014(94) | Ethiopia | 200 mother child pairs aged 6–23 months. | cross-sectional study | The main reason reported by the mothers for early initiation of complementary feeding was lack of knowledge. Mothers who have male child were three times more likely timely initiate complementary feeding than female child. |
| Senarath et al., 2012(95) | South Ashian countries | 19005 mother child pairs aged 6–23 months. | survey | The most consistent determinants of inappropriate complementary feeding practices across all countries were the low level of maternal education and household poverty. Limited exposure to media was also found to be a predictor for poor practices. •Inadequate antenatal care, untrained assistance at delivery and lack of post-natal contacts by health workers were among the health service factors that were associated with inappropriate feeding. |
| Senarath et al., 2012(96) | Sri Lanka | 2106 mother child pairs aged 6–23 months. | survey | Poor complementary feeding practices were associated with children living in the tea estate sector, lower maternal education, lower wealth index, shorter maternal height, lack of post-natal visits, unsatisfactory exposure to media and children with acute respiratory infections |
| Shaker-Berbari et al., 2021(97) | Middle East and North Africa region | 13821 mother child pairs aged 6–23 months. | survey | Maternal factors, including maternal education and age, household level factors such as paternal education and wealth, community-level factors (culture and geographic location), and utilization of health services, were associated with CF. |
| Shumey et al., 2013(98) | Ethiopia | 422 mother child pairs aged 6–23 months. | cross-sectional study | Educational level, occupation of mother, parity, having ANC follow up, and birth preparedness were found to be independent predictor of timely initiation of complementary feeding. |
| Sunuwar et al., 2023(99) | Nepal | 318 mother child pairs aged 6–23 months. | cross-sectional study | Multivariable analysis showed maternal characteristics such as mothers who gave birth at home and mothers in unpaid employment were associated with increased odds of inappropriate child feeding practices. Household economy (i.e. family with < 150 USD monthly income) was also associated with increased odds of inappropriate child feeding practices. |
| Supthanasup et al., 2022(100) | Thailand | 4125 mother child pairs aged 6–23 months. | survey | The proportion of inappropriate CF practices was higher among children living with caregivers other than their mothers. While maternal education and household income were positively associated with MDD and MAD, children of mothers from middleclass households consumed more sweetened beverages. |
| Tadesse et al., 2023(101) | Ethiopia | 482 mother child pairs aged 6–23 months. | Case-control | Part working situation of mother was negatively associated with inappropriate complementary feeding. Having no post-natal care visit, poor wealth status, food in-security, home delivery, having poor knowledge on infant and young child feeding, having no health education on complementary feeding and father’s job were found to be positively associated with inappropriate complementary feeding. |
| Tromp et al., 2013(102) | Netherlands | 3561 mother child pairs aged 6–23 months. | cohort | Determinants for very early introduction were being a single parent and infant day care attendance, young maternal age, multiple parities, no infant family history of asthma, atopy and no infant history of allergy to cow's milk, not fully breastfeeding for 4 months. |
| Umugwaneza., 2021(103) | Rwanda | 65 mother child pairs aged 6–23 months. | mixed method | Caregivers’ knowledge and beliefs about the benefits timely introduction of complementary food. At the society level, poverty in rural agrarian households was a barrier to optimal feeding practices. |
| Victor et al., 2014(104) | Tanzania | 2402 mother child pairs aged 6–23 months. | survey | Results from multivariate analyses indicated that the main risk factors for inappropriate complementary feeding practices in Tanzania include young child’s age (6–11 months), lower level of paternal/maternal education, limited access to mass media, lack of post-natal check-ups, and poor economic status. Overall, complementary feeding practices in Tanzania, as measured by dietary diversity, meal frequency and acceptable diet, are not adequately met, and there is a need for interventions to improve the nutritional status of young children in Tanzania. |
| Walters et al., 2019(105) | Malawi | 2294 mother child pairs aged 6–23 months. | survey | Maternal characteristics were significantly associated with optimal CF indicators. |
| Wasihun et al., 2024(106) | Ethiopia | 409 mother child pairs aged 6–23 months. | survey | Factors associated with -early initiation included place of residence, husband’s educational status, maternal occupation, number of antenatal care (ANC) visits, initial breastfeeding time, and medical illness. . |
| Yeshaneh et al., 2021(107) | Ethiopia | 264 mother child pairs aged 6–23 months. | cross-sectional study | Child age and having harmful culture on complementary feeding were found to be the two predictors of the complementary feeding practice of IDP mothers in the study area. |
| Yunitasari et al., 2022(108) | Indonesia | 502800 mother child pairs aged 6–23 months. | cross-sectional study | The probability of achieving minimum dietary diversity (MDD) was high in the following: children aged 18–23 months confidence interval, children of mothers with higher education, children from households with upper wealth index, children of mothers who received childbirth assistance by professionals, and children of mothers who had access to the Internet. |

1. Abate AD, Hassen SL, Temesgen MM. Timely initiation of complementary feeding practices and associated factors among children aged 6-23 months in Dessie Zuria District, Northeast Ethiopia: a community-based cross-sectional study. Front Pediatr. 2023;11:1062251.

2. Abate MW, Nigat AB, Demelash AT, Emiru TD, Tibebu NS, Tiruneh CM, et al. Prevalence of timely complementary feeding initiation and associated factors among mothers having children aged 6-24 months in rural north-central Ethiopia: Community based cross-sectional study. PloS One. 2022;17(5):e0267008.

3. Abebe H, Gashu M, Kebede A, Abata H, Yeshaneh A, Workye H, et al. Minimum acceptable diet and associated factors among children aged 6-23 months in Ethiopia. Ital J Pediatr. 2021 Oct 30;47(1):215.

4. Aber H, Kisakye AN, Babirye JN. Adherence to complementary feeding guidelines among caregivers of children aged 6-23 months in Lamwo district, rural Uganda. Pan Afr Med J. 2018;31:17.

5. Aga JA, Naupal-Forcadilla RT, Cayetano AC. Caregivers’ knowledge, attitude, and practices on complementary feeding of young children aged 6-23 months in Naga City, Philippines. J Hum Ecol Sustain. 2024;2(1):11.

6. Ahmad A, Madanijah S, Dwiriani CM, Kolopaking R. Determinant Factors of Maternal Knowledge on Appropriate Complementary Feeding of Children Aged 6-23 Months in Aceh. J Nutr Sci Vitaminol (Tokyo). 2020;66(Supplement):S239–43.

7. Ahmad I, Khalique N, Khalil S, Urfi null, Maroof M. Complementary feeding practices among children aged 6-23 months in Aligarh, Uttar Pradesh. J Fam Med Prim Care. 2017;6(2):386–91.

8. Ahmed JA, Sadeta KK, Lembo KH. Complementary Feeding Practices and Household Food Insecurity Status of Children Aged 6-23 Months in Shashemene City West Arsi Zone, Oromia, Ethiopia. Nurs Res Pract. 2022;2022:9387031.

9. Ahmed JA, Sadeta KK, Lenbo KH. Magnitude and factors associated with appropriate complementary feeding practice among mothers of children 6-23 months age in Shashemene town, Oromia- Ethiopia: Community based cross sectional study. PloS One. 2022;17(3):e0265716.

10. Akanbonga S, Hasan T, Chowdhury U, Kaiser A, Akter Bonny F, Lim IE, et al. Infant and young child feeding practices and associated socioeconomic and demographic factors among children aged 6–23 months in Ghana: Findings from Ghana Multiple Indicator Cluster Survey, 2017–2018. Plos One. 2023;18(6):e0286055.

11. Akpaki K, Galibois I, Blaney S. Feeding practices and factors associated with the provision of iron-rich foods to children aged 6–23 months in Matam area, Senegal. Public Health Nutr. 2021;24(14):4442–53.

12. Al-Samarrai MAM, Yaseen SM, Jadoo SAA. Knowledge, attitude, and practice of mothers about complementary feeding for infants aged 6-12 months in Anbar Province, Iraq. J Ideas Health. 2020;3(1):125–9.

13. Ali M, Arif M, Shah AA. Complementary feeding practices and associated factors among children aged 6-23 months in Pakistan. PloS One. 2021;16(2):e0247602.

14. Almeida MAM, Corrente JE, Vidal EI de O, Gomes C de B, Rinaldi AEM, Carvalhaes MA de BL. Patterns of complementary feeding introduction and associated factors in a cohort of Brazilian infants. BMC Pediatr. 2024 Oct 2;24(1):629.

15. Alzaheb RA. Factors Associated with the Early Introduction of Complementary Feeding in Saudi Arabia. Int J Environ Res Public Health. 2016 Jul 12;13(7):702.

16. Ariff S, Saddiq K, Khalid J, Sikanderali L, Tariq B, Shaheen F, et al. Determinants of infant and young complementary feeding practices among children 6-23 months of age in urban Pakistan: a multicenter longitudinal study. BMC Nutr. 2020 Dec 16;6(1):75.

17. Ayu EG, Gemebo TD, Nane D, Kuche AD, Dake SK. Inappropriate complementary feeding practice and associated factors among children aged 6-23 months in Shashemene, Southern Ethiopia: a community-based cross-sectional study. BMC Pediatr. 2024 Sep 10;24(1):573.

18. Ara R, Dipti T, Uddin M, Ali M, Rahman L. Feeding Practices and its Impact on Nutritional Status Children Under 2 Years in a Selected Rural Community of Bangladesh. J Armed Forces Med Coll Bangladesh. 2013 Sep 6;8(2):26–31.

19. Bably MB, Laditka SB, Mehta A, Ghosh-Jerath S, Racine EF. Timing and factors associated with complementary feeding in India. Health Care Women Int. 2023 Mar;44(3):220–33.

20. Belete S, Kebede N, Chane T, Melese W, Tadesse SE. Optimal complementary feeding practices and associated factors among mothers having children 6 to 23 months, south WOLLO zone, Dessie ZURIA, Ethiopia. J Pediatr Nurs. 2022;67:e106–12.

21. Berhanu Z, Alemu T, Argaw D. Predictors of inappropriate complementary feeding practice among children aged 6 to 23 months in Wonago District, South Ethiopia, 2017; case control study. BMC Pediatr. 2019 Dec;19(1):146.

22. Birhanu M, Abegaz T, Fikre R. Magnitude and Factors Associated with Optimal Complementary Feeding Practices among Children Aged 6-23 Months in Bensa District, Sidama Zone, South Ethiopia. Ethiop J Health Sci. 2019 Mar;29(2):153–64.

23. Bodjrènou FSU, Amoussa Hounkpatin W, Termote C, Dato G, Savy M. Determining factors associated with breastfeeding and complementary feeding practices in rural Southern Benin. Food Sci Nutr. 2021 Jan;9(1):135–44.

24. Bwalya R, Chama-Chiliba CM, Malinga S, Chirwa T. Association between household food security and infant feeding practices among women with children aged 6–23 months in rural Zambia. Plos One. 2023;18(10):e0292052.

25. Chane T, Bitew S, Mekonnen T, Fekadu W. Initiation of complementary feeding and associated factors among children of age 6-23 months in Sodo town, Southern Ethiopia: Cross-sectional study. Pediatr Rep. 2017 Nov 21;9(4):7240.

26. Chapagain RH. Factors affecting complementary feeding practices of Nepali mothers for 6 months to 24 months children. J Nepal Health Res Counc. 2013 May;11(24):205–7.

27. Dagne AH, Anteneh KT, Badi MB, Adhanu HH, Ahunie MA, Tebeje HD, et al. Appropriate complementary feeding practice and associated factors among mothers having children aged 6-24 months in Debre Tabor Hospital, North West Ethiopia, 2016. BMC Res Notes. 2019 Apr 8;12(1):215.

28. Debessa T, Befkadu Z, Darge T, Mitiku A, Negera E. Commercial complementary food feeding and associated factors among mothers of children aged 6-23 months old in Mettu Town, Southwest Ethiopia, 2022. BMC Nutr. 2023 Oct 24;9(1):118.

29. Dejene Y, Mezgebu GS, Tadesse SE. Minimum acceptable diet and its associated factors among children aged 6-23 months in Lalibela, northeast Ethiopia: a community-based cross-sectional study. J Nutr Sci. 2023;12:e41.

30. Derseh NM, Shewaye DA, Agimas MC, Alemayehu MA, Aragaw FM. Spatial variation and determinants of inappropriate complementary feeding practice and its effect on the undernutrition of infants and young children aged 6 to 23 months in Ethiopia by using the Ethiopian Mini-demographic and health survey, 2019: spatial and multilevel analysis. Front Public Health. 2023;11:1158397.

31. Dhami MV, Ogbo FA, Diallo TMO, Olusanya BO, Goson PC, Agho KE, et al. Infant and Young Child Feeding Practices among Adolescent Mothers and Associated Factors in India. Nutrients. 2021 Jul 12;13(7):2376.

32. Dhami MV, Ogbo FA, Osuagwu UL, Agho KE. Prevalence and factors associated with complementary feeding practices among children aged 6–23 months in India: a regional analysis. BMC Public Health. 2019 Dec;19(1):1034.

33. Dou N, Shakya E, Ngoutane RM, Garnier D, Kouame OR, Dain AL, et al. Promising trends and influencing factors of complementary feeding practices in Côte d’Ivoire: An analysis of nationally representative survey data between 1994 and 2016. Matern Child Nutr. 2023 Jan;19(1):e13418.

34. Duan Y, Yang Z, Lai J, Yu D, Chang S, Pang X, et al. Exclusive Breastfeeding Rate and Complementary Feeding Indicators in China: A National Representative Survey in 2013. Nutrients. 2018 Feb 22;10(2):249.

35. Dusingizimana T, Weber JL, Ramilan T, Iversen PO, Brough L. A qualitative analysis of infant and young child feeding practices in rural Rwanda. Public Health Nutr. 2021 Aug;24(12):3592–601.

36. Epheson B, Birhanu Z, Tamiru D, Feyissa GT. Complementary feeding practices and associated factors in Damot Weydie District, Welayta zone, South Ethiopia. BMC Public Health. 2018 Mar 27;18(1):419.

37. Erasmus CR, Pillay T, Siwela M. Factors affecting the choices made by primary caregivers during the complementary feeding transition period, KwaZulu-Natal, South Africa. South Afr J Clin Nutr. 2023;36(1):1–7.

38. Esan DT, Adegbilero-Iwari OE, Hussaini A, Adetunji AJ. Complementary feeding pattern and its determinants among mothers in selected primary health centers in the urban metropolis of Ekiti State, Nigeria. Sci Rep. 2022;12(1):6252.

39. Fanta M, Cherie HA. Magnitude and determinants of appropriate complementary feeding practice among mothers of children age 6-23 months in Western Ethiopia. PloS One. 2020;15(12):e0244277.

40. Gebretsadik MT, Adugna DT, Aliyu AD, Belachew T. Optimal complementary feeding practices of children aged 6-23 months in three agro-ecological rural districts of Jimma zones of southwest Ethiopia. J Nutr Sci. 2023;12:e40.

41. Gilano G, Sako S, Gilano K. Determinants of timely initiation of complementary feeding among children aged 6-23 months in Ethiopia. Sci Rep. 2022 Nov 9;12(1):19069.

42. Gizaw AT, Sopory P, Sudhakar M. Determinants of knowledge, attitude and self-efficacy towards complementary feeding among rural mothers: Baseline data of a cluster-randomized control trial in South West Ethiopia. PloS One. 2023;18(11):e0293267.

43. Goudet SM, Faiz S, Bogin BA, Griffiths PL. Pregnant women’s and community health workers’ perceptions of root causes of malnutrition among infants and young children in the slums of Dhaka, Bangladesh. Am J Public Health. 2011 Jul;101(7):1225–33.

44. Gurung TB, Paudel R, K C A, Acharya A, Khanal PK. Appropriate complementary feeding practice and associated factors among mothers of children aged 6-23 months in Bhimphedi rural municipality of Nepal. PloS One. 2024;19(3):e0299969.

45. Haile D, Belachew T, Berhanu G, Setegn T, Biadgilign S. Complementary feeding practices and associated factors among HIV positive mothers in Southern Ethiopia. J Health Popul Nutr. 2015 May 1;34:5.

46. Heidkamp RA, Ayoya MA, Teta IN, Stoltzfus RJ, Marhone JP. Complementary feeding practices and child growth outcomes in Haiti: an analysis of data from Demographic and Health Surveys. Matern Child Nutr. 2015 Oct;11(4):815–28.

47. Helle C, Hillesund ER, Øverby NC. Timing of complementary feeding and associations with maternal and infant characteristics: A Norwegian cross-sectional study. PloS One. 2018;13(6):e0199455.

48. Harvey S, Callaby J, Roberts L. <no title>. 2017 Jul 3;

49. Hazir T, Senarath U, Agho K, Akram DS, Kazmi N, Abbasi S, et al. Determinants of inappropriate timing of introducing solid, semi-solid or soft food to infants in Pakistan: secondary data analysis of Demographic and Health Survey 2006-2007. Matern Child Nutr. 2012 Jan;8 Suppl 1(Suppl 1):78–88.

50. Issaka AI, Agho KE, Burns P, Page A, Dibley MJ. Determinants of inadequate complementary feeding practices among children aged 6–23 months in Ghana. Public Health Nutr. 2015;18(4):669–78.

51. Issaka AI, Agho KE, Ezeh OK, Renzaho AM. Population-attributable risk estimates for factors associated with inappropriate complementary feeding practices in The Gambia. Public Health Nutr. 2017 Dec;20(17):3135–44.

52. Janmohamed A, Luvsanjamba M, Norov B, Batsaikhan E, Jamiyan B, Blankenship JL. Complementary feeding practices and associated factors among Mongolian children 6–23 months of age. Matern Child Nutr. 2020 Oct;16(S2):e12838.

53. Joshi N, Agho KE, Dibley MJ, Senarath U, Tiwari K. Determinants of inappropriate complementary feeding practices in young children in Nepal: secondary data analysis of Demographic and Health Survey 2006. Matern Child Nutr. 2012 Jan;8 Suppl 1(Suppl 1):45–59.

54. Jubayer A, Nowar A, Islam S, Islam MdH, Nayan MdM. Complementary feeding practices and their determinants among children aged 6–23 months in rural Bangladesh: evidence from Bangladesh Integrated Household Survey (BIHS) 2018–2019 evaluated against WHO/UNICEF guideline -2021. Arch Public Health. 2023 Jun 21;81(1):114.

55. Kabir A, Maitrot MRL. Factors influencing feeding practices of extreme poor infants and young children in families of working mothers in Dhaka slums: A qualitative study. PloS One. 2017;12(2):e0172119.

56. Kabir I, Khanam M, Agho KE, Mihrshahi S, Dibley MJ, Roy SK. Determinants of inappropriate complementary feeding practices in infant and young children in Bangladesh: secondary data analysis of Demographic Health Survey 2007. Matern Child Nutr. 2012 Jan;8 Suppl 1(Suppl 1):11–27.

57. Kambale RM, Ngaboyeka GA, Kasengi JB, Niyitegeka S, Cinkenye BR, Baruti A, et al. Minimum acceptable diet among children aged 6-23 months in South Kivu, Democratic Republic of Congo: a community-based cross-sectional study. BMC Pediatr. 2021 May 19;21(1):239.

58. Kassa T, Meshesha B, Haji Y, Ebrahim J. Appropriate complementary feeding practices and associated factors among mothers of children age 6–23 months in Southern Ethiopia, 2015. BMC Pediatr. 2016 Dec;16(1):131.

59. Kegne T, Alemu YM, Wassie GT. Timely initiation of complementary feeding and associated factors among mothers having children aged 6 to 24 months in North-West Ethiopia: a comparative cross-sectional study. BMC Pediatr. 2024 Jul 3;24(1):428.

60. Kinoti FN, Mutai C, Wanzala P, Karanja SM. Factors associated with infant feeding practices and nutritional status among children aged 6-24 months attending child welfare clinics in Kajiado Sub-County. East Afr Med J. 2016 Nov 14;93(8):348–53.

61. Kimiywe J, Chege PM. Complementary feeding practices and nutritional status of children 6-23 months in Kitui County, Kenya. J Appl Biosci. 2015 Feb 26;85:7881–90.

62. Khanal V, Sauer K, Zhao Y. Determinants of complementary feeding practices among Nepalese children aged 6-23 months: findings from Demographic and Health Survey 2011. BMC Pediatr. 2013 Aug 28;13:131.

63. Kurnia ID, Rachmawati PD, Arief YS, Krisnana I, Rithpho P, Arifin H. Factors associated with infant and young child feeding practices in children aged 6-23 months in Indonesia: A nationwide study. J Pediatr Nurs. 2024;78:82–8.

64. Liaqat P, Rizvi MA, Qayyum A, Ahmed H. Association between complementary feeding practice and mothers education status in Islamabad. J Hum Nutr Diet Off J Br Diet Assoc. 2007 Aug;20(4):340–4.

65. Liu J, Huo J, Sun J, Huang J, Gong W, Wang O. Prevalence of Complementary Feeding Indicators and Associated Factors Among 6- to 23-Month Breastfed Infants and Young Children in Poor Rural Areas of China. Front Public Health. 2021;9:691894.

66. Mphasha MH, Mokubela G, Ramokotedi T, Kgari T. Exploratory study on factors influencing the introduction of complementary feeding amongst caregivers of children between 6 and 24 months of age in Polokwane, Limpopo province. South Afr Fam Pract [Internet]. 2023 Apr 23 [cited 2025 Aug 6];65(1). Available from: https://www.ajol.info/index.php/safp/article/view/246301

67. Maciel B, Moraes ML, Soares AM, Cruz I, de Andrade M, Filho JQ, et al. Infant feeding practices and determinant variables for early complementary feeding in the first 8 months of life: results from the Brazilian MAL-ED cohort site. Public Health Nutr. 2018 Sep;21(13):2462–70.

68. Mamo ZB, Wudneh A, Molla W. Determinants of complementary feeding initiation time among 6–23 months children in Gedeo Zone, South Ethiopia: Community-based case-control study. Int J Afr Nurs Sci. 2022;16:100418.

69. Markos M, Samuel B, Challa A. Minimum acceptable diet and associated factors among 6-23 months old children enrolled in outpatient therapeutic program in the Tulla district, Sidama region, Ethiopia: a community-based cross-sectional study. J Health Popul Nutr. 2024 Jul 8;43(1):106.

70. Martins FA, Ramalho AA, de Andrade AM, Opitz SP, Koifman RJ, de Aguiar DM, et al. Minimum acceptable diet in a cohort of children aged between 6 and 15 months: Complementary feeding assessment and associated factors in the Brazilian western Amazon. Nutr Burbank Los Angel Cty Calif. 2024 Jan;117:112231.

71. Mekonen EG, Zegeye AF, Workneh BS. Complementary feeding practices and associated factors among mothers of children aged 6 to 23 months in Sub-saharan African countries: a multilevel analysis of the recent demographic and health survey. BMC Public Health. 2024 Jan 8;24(1):115.

72. Mitchodigni IM, Amoussa Hounkpatin W, Ntandou-Bouzitou G, Avohou H, Termote C, Kennedy G, et al. Complementary feeding practices: determinants of dietary diversity and meal frequency among children aged 6–23 months in Southern Benin. Food Secur. 2017 Oct;9(5):1117–30.

73. Mokori A, Schonfeldt H, Hendriks SL. Child factors associated with complementary feeding practices in Uganda. South Afr J Clin Nutr. 2017 Mar 31;30(1):7–14.

74. Owino V, Amadi B, Sinkala M, Filteau S, Tomkins A. Complementary Feeding Practices And Nutrient Intake From Habitual Complementary Foods Of Infants And Children Aged 6-18 Months Old In Lusaka, Zambia. Afr J Food Agric Nutr Dev. 2008;8(1):28–47.

75. Okafoagu NC, Oche OM, Raji MO, Onankpa B, Raji I. Factors influencing complementary and weaning practices among women in rural communities of Sokoto state, Nigeria. Pan Afr Med J [Internet]. 2017 [cited 2025 Aug 6];28(1). Available from: https://www.ajol.info/index.php/pamj/article/view/167152

76. Na M, Aguayo VM, Arimond M, Stewart CP. Risk factors of poor complementary feeding practices in Pakistani children aged 6–23 months: A multilevel analysis of the Demographic and Health Survey 2012–2013. Matern Child Nutr. 2017 Oct;13(S2):e12463.

77. Na M, Aguayo VM, Arimond M, Mustaphi P, Stewart CP. Predictors of complementary feeding practices in Afghanistan: Analysis of the 2015 Demographic and Health Survey. Matern Child Nutr. 2018 Nov;14 Suppl 4(Suppl 4):e12696.

78. Na M, Aguayo VM, Arimond M, Narayan A, Stewart CP. Stagnating trends in complementary feeding practices in Bangladesh: An analysis of national surveys from 2004-2014. Matern Child Nutr. 2018 Nov;14 Suppl 4(Suppl 4):e12624.

79. Na M, Aguayo VM, Arimond M, Dahal P, Lamichhane B, Pokharel R, et al. Trends and predictors of appropriate complementary feeding practices in Nepal: An analysis of national household survey data collected between 2001 and 2014. Matern Child Nutr. 2018 Nov;14 Suppl 4(Suppl 4):e12564.

80. Ng CS, Dibley MJ, Agho KE. Complementary feeding indicators and determinants of poor feeding practices in Indonesia: a secondary analysis of 2007 Demographic and Health Survey data. Public Health Nutr. 2012 May;15(5):827–39.

81. Nkoka O, Mhone TG, Ntenda PAM. Factors associated with complementary feeding practices among children aged 6-23 mo in Malawi: an analysis of the Demographic and Health Survey 2015-2016. Int Health. 2018 Nov 1;10(6):466–79.

82. Ogbo FA, Ogeleka P, Awosemo AO. Trends and determinants of complementary feeding practices in Tanzania, 2004-2016. Trop Med Health. 2018;46:40.

83. Patel A, Pusdekar Y, Badhoniya N, Borkar J, Agho KE, Dibley MJ. Determinants of inappropriate complementary feeding practices in young children in India: secondary analysis of National Family Health Survey 2005-2006. Matern Child Nutr. 2012 Jan;8 Suppl 1(Suppl 1):28–44.

84. Pelto GH, Armar-Klemesu M. Balancing nurturance, cost and time: complementary feeding in Accra, Ghana. Matern Child Nutr. 2011 Oct;7 Suppl 3(Suppl 3):66–81.

85. Paul SK, Roy S, Islam QR, Islam MZ, Akteruzzaman M, Rouf MA, et al. Barriers of Appropriate Complementary Feeding Practices in Under 2 Children. J Bangladesh Coll Physicians Surg. 2016 Jun 8;33(4):195–201.

86. Rakotomanana H, Gates GE, Hildebrand D, Stoecker BJ. Situation and determinants of the infant and young child feeding (IYCF) indicators in Madagascar: analysis of the 2009 Demographic and Health Survey. BMC Public Health. 2017 Oct 16;17(1):812.

87. Rakotomanana H, Hildebrand D, Gates GE, Thomas DG, Fawbush F, Stoecker BJ. Maternal knowledge, attitudes, and practices of complementary feeding and child undernutrition in the Vakinankaratra Region of Madagascar: a mixed-methods study. Curr Dev Nutr. 2020;4(11):nzaa162.

88. Rebhan B, Kohlhuber M, Schwegler U, Koletzko BV, Fromme H. Infant feeding practices and associated factors through the first 9 months of life in Bavaria, Germany. J Pediatr Gastroenterol Nutr. 2009 Oct;49(4):467–73.

89. Reda EB, Teferra AS, Gebregziabher MG. Time to initiate complementary feeding and associated factors among mothers with children aged 6-24 months in Tahtay Maichew district, northern Ethiopia. BMC Res Notes. 2019 Jan 14;12(1):17.

90. Saaka M, Awini S, Nang E. Prevalence and predictors of appropriate complementary feeding practice among mothers with children 6–23 months in Northern Ghana. World Nutr. 2022;13(2):14–23.

91. Hoche S, Meshesha B, Wakgari N. Sub-optimal breastfeeding and its associated factors in rural communities of Hula District, southern Ethiopia: a cross-sectional study. Ethiop J Health Sci. 2018 Jan 10;28(1):49–62.

92. Samuel FO, Akintayo B, Eyinla TE. Complementary feeding knowledge and practices of caregivers in orphanages improved after nutrition education intervention in Ibadan, Nigeria. Open J Nurs. 2021;11(7):642–52.

93. Scarpa G, Berrang-Ford L, Twesigomwe S, Kakwangire P, Galazoula M, Zavaleta-Cortijo C, et al. Socio-economic and environmental factors affecting breastfeeding and complementary feeding practices among Batwa and Bakiga communities in south-western Uganda. PLOS Glob Public Health. 2022;2(3):e0000144.

94. Semahegn A, Tesfaye G, Bogale A. Complementary feeding practice of mothers and associated factors in Hiwot Fana Specialized Hospital, Eastern Ethiopia. Pan Afr Med J. 2014;18:143.

95. Senarath U, Agho KE, Akram D e S, Godakandage SSP, Hazir T, Jayawickrama H, et al. Comparisons of complementary feeding indicators and associated factors in children aged 6-23 months across five South Asian countries. Matern Child Nutr. 2012 Jan;8 Suppl 1(Suppl 1):89–106.

96. Senarath U, Godakandage SSP, Jayawickrama H, Siriwardena I, Dibley MJ. Determinants of inappropriate complementary feeding practices in young children in Sri Lanka: secondary data analysis of Demographic and Health Survey 2006-2007. Matern Child Nutr. 2012 Jan;8 Suppl 1(Suppl 1):60–77.

97. Shaker‐Berbari L, Qahoush Tyler V, Akik C, Jamaluddine Z, Ghattas H. Predictors of complementary feeding practices among children aged 6–23 months in five countries in the Middle East and North Africa region. Matern Child Nutr. 2021 Oct;17(4):e13223.

98. Shumey A, Demissie M, Berhane Y. Timely initiation of complementary feeding and associated factors among children aged 6 to 12 months in Northern Ethiopia: an institution-based cross-sectional study. BMC Public Health. 2013 Nov 6;13:1050.

99. Sunuwar DR, Bhatta A, Rai A, Chaudhary NK, Tamang MK, Nayaju S, et al. The factors influencing inappropriate child feeding practices among families receiving nutrition allowance in the Himalayan region of Nepal. BMC Nutr. 2023 Feb 20;9(1):33.

100. Supthanasup A, Cetthakrikul N, Kelly M, Sarma H, Banwell C. Determinants of complementary feeding indicators: a secondary analysis of Thailand multiple indicators cluster survey 2019. Nutrients. 2022;14(20):4370.

101. Tadesse M, Ali Dawed Y, Fentaw Z, Endawike A, Adamu K. Determinants of inappropriate complementary feeding among children 6–23 months of age in Dessie City Northeast Ethiopia: a case-control study. BMC Nutr. 2023 Nov 3;9(1):124.

102. Tromp IIM, Briedé S, Kiefte-de Jong JC, Renders CM, Jaddoe VWV, Franco OH, et al. Factors associated with the timing of introduction of complementary feeding: the Generation R Study. Eur J Clin Nutr. 2013 Jun;67(6):625–30.

103. Umugwaneza M, Havemann-Nel L, Vorster HH, Wentzel-Viljoen E. Factors influencing complementary feeding practices in rural and semi-urban Rwanda: a qualitative study. J Nutr Sci. 2021 Jan;10:e45.

104. Victor R, Baines SK, Agho KE, Dibley MJ. Factors associated with inappropriate complementary feeding practices among children aged 6–23 months in T anzania. Matern Child Nutr. 2014 Oct;10(4):545–61.

105. Walters CN, Rakotomanana H, Komakech JJ, Stoecker BJ. Maternal determinants of optimal breastfeeding and complementary feeding and their association with child undernutrition in Malawi (2015–2016). BMC Public Health. 2019 Dec;19(1):1503.

106. Wasihun Y, Addissie G, Yigezu M, Kebede N. Early initiation of complementary feeding practice and its associated factors among children aged 6 to 24 months in Northeast Ethiopia. J Health Popul Nutr. 2024 May 16;43(1):67.

107. Yeshaneh A, Zebene M, Gashu M, Abebe H, Abate H. Complementary feeding practice and associated factors among internally displaced mothers of children aged 6–23 months in Amhara region, Northwest Ethiopia: a cross-sectional study. BMC Pediatr. 2021 Dec;21(1):583.

108. Yunitasari E, Al Faisal AH, Efendi F, Kusumaningrum T, Yunita FC, Chong MC. Factors associated with complementary feeding practices among children aged 6–23 months in Indonesia. BMC Pediatr. 2022 Dec 21;22(1):727.
